# Inhibition of Raf-MEK-ERK and Hypoxia pathways by *Phyllanthus* prevents metastasis in human lung (A549) cancer cell line

**DOI:** 10.1186/1472-6882-13-271

**Published:** 2013-10-20

**Authors:** Sau Har Lee, Indu Bala Jaganath, Rishya Manikam, Shamala Devi Sekaran

**Affiliations:** 1 Department of Medical Microbiology, Faculty of Medicine, Universiti Malaya, Kuala Lumpur, Malaysia; 2 Biotechnology Centre, Malaysia Agricultural Research and Development Institute (MARDI), Serdang, Malaysia; 3 Trauma & Emergency Department, University Malaya Medical Centre, Kuala Lumpur, Malaysia

**Keywords:** *Phyllanthus*, Metastasis, Apoptosis, ERK1/2, HIF-1α

## Abstract

**Background:**

Lung cancer constitutes one of the malignancies with the greatest incidence and mortality rates with 1.6 million new cases and 1.4 million deaths each year. Prognosis remains poor due to deleterious development of multidrug resistance resulting in less than 15% lung cancer patients reaching five years survival. We have previously shown that *Phyllanthus* induced apoptosis in conjunction with its antimetastastic action. In the current study, we aimed to determine the signaling pathways utilized by *Phyllanthus* to exert its antimetastatic activities.

**Methods:**

Cancer 10-pathway reporter array was performed to screen the pathways affected by *Phyllanthus* in lung carcinoma cell line (A549) to exert its antimetastatic effects. Results from this array were then confirmed with western blotting, cell cycle analysis, zymography technique, and cell based ELISA assay for human total iNOS. Two-dimensional gel electrophoresis was subsequently carried out to study the differential protein expressions in A549 after treatment with *Phyllanthus*.

**Results:**

*Phyllanthus* was observed to cause antimetastatic activities by inhibiting ERK1/2 pathway via suppression of Raf protein. Inhibition of this pathway resulted in the suppression of MMP2, MMP7, and MMP9 expression to stop A549 metastasis. *Phyllanthus* also inhibits hypoxia pathway via inhibition of HIF-1α that led to reduced VEGF and iNOS expressions. Proteomic analysis revealed a number of proteins downregulated by *Phyllanthus* that were involved in metastatic processes, including invasion and mobility proteins (cytoskeletal proteins), transcriptional proteins (proliferating cell nuclear antigen; zinc finger protein), antiapoptotic protein (Bcl2) and various glycolytic enzymes. Among the four *Phyllanthus* species tested, *P. urinaria* showed the greatest antimetastatic activity.

**Conclusions:**

*Phyllanthus* inhibits A549 metastasis by suppressing ERK1/2 and hypoxia pathways that led to suppression of various critical proteins for A549 invasion and migration.

## Background

Lung cancer constitutes one of the malignancies with the greatest incidence and mortality rates with 1.6 million new cases and 1.4 million deaths each year [1,2]. Although initial use of platin-based cytotoxic chemotherapy improved the overall survival rate and life quality of the patients, the outcome remains poor due to deleterious development of drug resistance resulting in less than 15% lung cancer patients surviving for at least five years [2,3]. Approximately 80 - 85% of the lung cancer patients are diagnosed with an advanced stage of non-small cell lung cancer (NSCLC) that has limited therapeutic options due to metastasis [1,4,5] and among the three types of NSCLC, patients identified with squamous carcinoma which involves lymph node metastasis accounts for 60% [5,6]. In the advance stage of NSCLC, surgery is not possible to remove all apparent lesions hence leading to the high rate of cancer recurrence [7]. Therefore, lethality of lung cancer is often attributed to late diagnosis, metastasis, and the occurrence of drug resistance [8]. Thus, the development of an efficient cancer diagnostic method is crucial since an early detection of cancer while it is still localized and curable is one of the most promising approaches to reduce increasing cancer burden [9]. This requires an understanding of the pathophysiology of the disease [10,11] which can be made possible via proteomic studies. Cancer proteomics allow the screening of early diagnostic markers or potential drug targets [6] by comparing the proteomes of the diseased and diseased-treated samples that allows identification of aberrantly expressed proteins [5,10]. These proteins could be biomarker candidates to expedite non-invasive diagnosis of early-stage malignant tumor, as well as to aid the monitoring of tumor progression and therapy effectiveness [12]. Similar to other malignant pathologies, tumor markers for NSCLC remained inadequate. Serum biomarkers employed in the current clinical setting such as ENO (enolase alpha), CEA (carcinoembryonic antigen), SCC (squamous cell carcinoma), CA-125 (cancer antigen 125) or TPA (tissue polypeptide antigen) are not satisfactory due to their low sensitivity and specificity [13,14]. In the quest for novel diagnostic or prognostic biomarkers, the proteomic technique is ideal since it permits qualitative and quantitative analysis of numerous proteins simultaneously [9,13]. Moreover, the search for biomarkers at the protein level is more dependable than at the transcriptional level as protein expression may not necessarily associate with mRNA expression [14].

Natural-product based drugs are gaining their popularity as preventive medicines or for health management, and hence this has spurred an intensive search for bioactive plant-derived anticancer compounds [15]. The genus *Phyllanthus* is one of the most widely distributed plants throughout the Amazon rainforests as well as other tropical and subtropical regions. Abundant studies on *Phyllanthus spp*. started in the late 1980′s when the clinical efficacy of *Phyllanthus niruri* against viral Hepatitis B was observed [16]. Various therapeutic actions of this genus have been reported, including being antihepatotoxic, antilithic, antihypertensive, anticarcinogenic, and most recently anti-HIV as well [16-19]. We have demonstrated antimetastatic and antiproliferative activities of *Phyllanthus* against several metastatic cell lines such as lung carcinoma (A549), breast (MCF-7) carcinomas [17], melanoma (MeWo), as well as prostate (PC-3) carcinoma [20].

Nevertheless, the exact mechanisms for the antimetastatic activities of *Phyllanthus* are still uncertain. MAP kinase (MAPK) is one of the signaling pathways known to mediate metastasis via transmission of extracellular stimuli into the nucleus that activate the serine/threonine kinases which belong to the MAPK superfamily [21]. MAPK serine/threonine superfamily is made up of three well-described subgroups, including ERK1/2 or p44/42 MAPK, JNK/SAPK, and p38 MAPK. These diverse pathways with distinct downstream targets are activated by various stimuli to regulate cell proliferation, apoptosis, and metastasis [21,22]. Regulation of metastasis by MAPK is mainly via controlling the cells’ expression of matrix metalloproteinases (MMPs) enzymes by decreasing the nuclear levels of NFκB, c-Fos, or c-Jun [21,22]. The MMPs are a family of at least 20 members of highly homologous, zinc- and calcium-dependent endopeptidases which can degrade virtually all extracellular matrix (ECM) components [23]. Among these MMPs, MMP2 and MMP9 are abundantly expressed, secreted, and activated in various malignant tumors [22,24]. Recent studies also shown that MMP7 plays an essential role in ectodomain shedding of cell-surface molecules such as EGFR, HB-EGF, Fas ligand, and E-cadherin in addition to its ability to degrade ECM components. Therefore, these enzymes are the most vital ones implicated in cancer invasion and metastasis [24,25].

Hypoxia pathway is another chief regulator of metastatic process. Hypoxia-inducible factor-1 (HIF-1) is the determining factor for oxygen-dependent gene regulation and is a dimer of HIF-1α and HIF-1β. Its regulation is mainly governed by the transactivation and stabilization of HIF-1α protein resided in the cytoplasm [26]. Under normoxia condition, HIF-1α instantaneously interacts with von Hipple–Lindau (pVHL) ubiquitin E3 ligase complex and is targeted for proteasomal degradation due to hydroxylation of two specific proline residues within the oxygen-dependent degradation domain by prolyl hydroxylases [7,26]. Contrarily, HIF-1α will be stabilized with increased expression during hypoxic condition, allowing it to translocate into the nucleus and dimerize with HIF-1β. HIF-1 will subsequently bind to the hypoxia response elements to regulate transcription of over 100 target genes which favors tumor growth and metastasis, including vascular endothelial growth factor (VEGF) as well as genes that regulate cellular processes such as energy metabolism, cell proliferation, vascular development and remodelling, as well as vasotone [2,7,26].

In our previous study, *Phyllanthus* extracts were shown to inhibit A549 (lung carcinoma) cells growth with IC_50_ values ranging from 60–130 μg/ml and 200–470 μg/ml for methanolic and aqueous extracts respectively [17]. We also demonstrated that they effectively reduced invasion, migration, and adhesion activities of A549 cells in a dose-dependent manner and was capable of inducing apoptosis along with its antimetastastic action [17]. Therefore, the main objective of this study was to determine the protein expression profile of *Phyllanthus*-treated A549 cells and how the signaling pathways were utilized by *Phyllanthus* in order to exert its antiproliferative and antimetastatic activities on this cell line.

## Methods

### Plant extracts and standard drugs

The crude extracts (aqueous and methanolic) of each *Phyllanthus spp*., namely *P. niruri*, *P. urinaria*, *P. watsonii* and *P. amarus*, were obtained from the Malaysian Agriculture and Research Development Institute (MARDI), Malaysia. The aqueous extracts were prepared by dissolving 10 mg in 1ml of sterile PBS (Final concentration 10 mg/ml), whereas, the methanolic extracts were prepared by dissolving 40 mg in 1ml of DMSO (Final concentration 40 mg/ml). The tubes containing the extracts were wrapped with aluminium foil and stored at -20°C until use. A single batch of extracts was used for all the experiments. The IC_50_ concentrations that were used as one of the treatment conditions in most of the experimental assays (Table 1) as well as the polyphenol contents of these extracts have been determined and published in the previous study [17]. Cisplatin and Doxorubicin (MERCK – 1 mg/ml) as standard anticancer drugs for lung and breast carcinomas respectively were included as the controls.

**Table 1:** **Cytotoxic effect [IC**_**50 **_**(μg/ml)] of *Phyllanthus *** **extracts against A549 and MCF-7**

			**IC**_**50 **_**(μg/ml) ± SEM**	
			**Cancer cell lines**	
		**Solvents**	**A549**	**MCF-7**
Plant extracts	*P. niruri (P.n)*	Aqueous	466.7 ± 41.63	179.7 ± 0.58
		Methanolic	128.3 ± 17.56	62.3 ± 9.07
	*P. urinaria (P.u)*	Aqueous	215.0 ± 21.79	139.3 ± 1.16
		Methanolic	69.0 ± 11.53	48.7 ± 10.02
	*P. watsonii (P.w)*	Aqueous	198.3 ± 10.41	104.0 ± 10.39
		Methanolic	61.3 ± 16.17	49.0 ± 8.19
	*P. amarus (P.a)*	Aqueous	240.0 ± 26.46	156.7 ± 5.77
		Methanolic	126.7 ± 7.64	56.3 ± 6.66

### Cell culture

Human lung carcinoma (A549) cell line was purchased from American Type Culture Collection (ATCC, USA) and was grown in RPMI-1640 (Roswell Park Memorial Institute). To ensure growth and viability of the cells, the mediums were supplemented with 10% FBS (Gibco) and incubated in a humidified atmosphere with 5% CO_2_ at 37°C.

### Transient transfection and cancer 10-pathway reporter array

Cancer is a group of diseases strongly correlated with defects in signal transduction proteins. Various key signaling pathways have been implicated in human tumorigenesis. Analysis of these signaling pathways was performed using Cignal Finder Cancer 10-pathway Reporter Array kit (SABiosciences, QIAGEN, USA). Transient transfection was performed using TransIT-LT1 (Mirus Bio, USA). Both plasmid DNAs for the respective signaling pathways provided in the kit as well as TransIT-LT1 were diluted using Opti-MEM I reduced serum medium (Invitrogen, USA). After that, plasmid DNAs and the TransIT-LT1 was mixed to allow TransIT-LT1/DNA complex formation. Subsequently, 90 μl (approximately 10000 cells) of the cell suspension were mixed with 10 μl of the complex and added into the designated wells of a 96-well cell culture white microplate (Nunc, Thermo Fisher Scientific, USA). The culture plate was rocked for 5 minutes on a rocker before it was incubated in a 5% CO_2_ incubator at 37ºC overnight. After the transfection of cells with various plasmid DNAs for the respective signaling pathways, the cells were incubated with different *Phyllanthus* extracts for another 24 hours. Dual-Glo Luciferase reagent was then added into each well and incubated at room temperature for 10 minutes before reading the firefly luminescence generated using the GloMax Multi Detection System (Promega, USA). This was followed by the addition of Dual-Glo Stop & Glo reagent to all wells. Similarly, another renilla luminescence reading was obtained after the plate was incubated for another 10 minutes. The firefly constructs monitor changes in the activity of a key transcription factor which is a downstream target of a particular signaling pathway. Meanwhile, renilla construct acts as an internal control for transfection efficiencies normalization as well as to monitor cell viability. Luminescence for each wells were determined by calculating the ratio of its firefly to renilla luminescence.

### Cell cycle analysis

DNA content analysis is performed by Propidium iodide (PI) staining which binds to the distinct amount of DNA content in the cells at different phases of the cell cycle. Briefly, cells were seeded at 10^5^ cells/well, treated with extracts at their IC_50_ values, and incubated at various time periods from 0 to 72 hours. At the end of each incubation period, cells treated with or without *Phyllanthus* extracts were harvested and fixed with ice-cold 70% ethanol for at least 1 hour at -20ºC. Cells were then pelleted, washed once with PBS, resuspended in the PI solution [10μg/ml PI (Sigma) and 1mg/ml RNase A in PBS], and incubated in a 37°C water bath for 30 minutes. Data acquisition was performed using a Becton Dickinson FACSCalibur flow cytometer and CellQuest software and subsequently analysed using WinMDI 2.9 software. The distribution of cell percentages in each cell cycle phase is determined by setting gates based on their amount of DNA content.

### Preparation of cytoplasmic protein lysate

Protein lysates were prepared for western blotting and proteomic analysis for both *Phyllanthus*-treated and untreated samples. Cells treated with *Phyllanthus* extracts were detached from the culture plates using 0.5% trypsin-EDTA and washed twice with phosphate buffered saline (PBS), centrifuging at 1500 rpm for 5 minutes. Two hundred microliters of lysis buffer (7M urea, 2M thiourea, 4% CHAPS, 2% IPG buffer, 40 mM DTT) was added to the cell pellet and incubated on ice for 30 minutes before collecting the supernatant into a new 1.5 ml tube followed by addition of 4× sample volume of iced-cold acetone. After that, the sample was incubated at -20°C overnight and centrifuged at 14000 rpm for 15 minutes at 4°C. After centrifugation, the supernatant was discarded while the cytoplasmic protein pellet was diluted in 150 μl of rehydration buffer (7 M urea, 2 M thiourea, 2% CHAPS, 0.5% IPG Buffer, 0.002% bromophenol blue). Cytoplasmic protein lysate was either stored at -80°C for storage or quantified immediately for subsequent experiments.

### Western blot assay

Protein lysate concentration was determined using 2-D Quant kit (GE Healthcare, USA) according to the manufacturer’s instructions. For one-dimensional western blotting, thirty micrograms protein of each samples were mixed with 4× sample buffer (50mM Tris-HCl [pH6.8], 2% SDS, 10% glycerol, 1% β-mercaptoethanol, 12.5mM EDTA, 0.02% bromophenol blue) before they were loaded on a 12.5% of SDS-polyacrylamide gel. The proteins were separated at 100V for approximately 1 hour. Alternatively, one hundred and fifty micrograms for each protein samples were separated by two-dimensional (2D) gel electrophoresis method using 7 cm IPG gel strips with pH 3–11 NL range (GE Healthcare, USA). After electrophoresis, the stack or sandwich was assembled and the proteins were transferred onto a nitrocellulose membrane (GE Healthcare, USA) at 250 mA for 1 hour. After the transfer, the membrane was blocked with Tris buffered saline buffer consisting 0.1% Tween 20 (TBST) and 5% dry milk. Subsequently, membranes were incubated with various primary antibodies and secondary antibodies diluted using blocking solution. The immune-reacted proteins were detected via chromogenic method by addition of DAB substrate onto the membrane to form a protein band. Anti-pan-Ras, anti-c-Raf, anti-c-Myc, anti-Bcl-2, anti-Hif-1α, anti-c-Jun/AP-1, anti-p53, anti-Elk1, anti-JNK1/2, anti-VEGF, goat anti-mouse IgG peroxidase conjugate, and goat anti-rabbit IgG peroxidase conjugate antibodies were purchased from Merck Millipore, Germany while anti-RSK antibody was purchased from Thermo Fischer Scientific, USA. These antibodies were chosen based on their essential roles in the pathways modulated by *Phyllanthus* as detected using the Cignal Finder Cancer 10-pathway Reporter Array. A p53 antibody was included to determine any involvement of p53 pathway in response to *Phyllanthus* treatment.

### Zymography assay

Zymography is a simple and sensitive technique employed to study extracellular matrix-degrading proteases such as MMPs based on their substrate specificity and molecular weight. Briefly, cells were seeded at 1 × 10^5^ cells/well in a 24-well microtiter plate and treated with increasing concentrations of aqueous (50 μg/ml, IC_50_, 500 μg/ml) and methanolic (20 μg/ml, IC_50_, 200 μg/ml) extracts. After 72 hours incubation, supernatants were collected and stored at -20°C to be used as conditioned media. The conditioned media was mixed with 2× sample buffer (0.5M Tris-HCl [pH6.8], 87% glycerol, 10% SDS, 0.1% bromophenol blue) and subsequently loaded onto a 12.5% SDS-polyacrylamide gels that had been copolymerized with 0.1% gelatin or 0.2% casein. The gel was then run at approximately 125V for about 60 minutes. When the proteins were completely resolved, the gel was washed twice with renaturing buffer (2.5% Triton X-100) on a shaker at room temperature, 1 hour for each washing. Next, the gel was incubated with developing buffer (12.1 g Tris, 63 g Tris-HCl, 117 g NaCl, 7.4 g CaCl_2_, 0.2% Brij35, 1L distilled water) overnight at 37°C before it was stained with 0.1% Coomasie blue for 1 hour. Finally, the gel was destained with destaining solution and the presence of matrix metalloproteinase enzyme was indicated as an opaque, unstained band against a dark blue background.

### Human total iNOS and GADPH immunoassay

Inducible Nitric Oxide Synthase (iNOS) is often upregulated in tumor cells and is an important regulator for vascularization and angiogenesis. In order to measure the total iNOS in whole cell, a cell-based ELISA, Human Total iNOS Immunoassay (R&D Systems, USA) was performed according to the manufacturer’s instructions.

### 2-Dimensional gel electrophoresis

Five hundred microgram proteins for A549 cells (treated and untreated samples) were rehydrated overnight on 13 cm IPG gel strips with pH 3–11 NL range (GE Healthcare, USA). Strips rehydrated with proteins were then transferred into IPG chambers and focused using Ettan IPGphor Isoelectric Focusing unit (GE Healthcare, USA). Before proceeding with SDS-PAGE, the strips were first subjected to a two-step equilibration procedure for 15 minutes each. Firstly, they were equilibrated with an SDS-PAGE equilibration base buffer added with 2% (w/v) Dithiothreitol (DTT), followed by equilibration with 2.5% (w/v) Iodoacetamide (IAA). The strips were then placed onto a 12.5% SDS-PAGE gel and sealed with agarose sealing solution. Second dimensional separation was carried out using Ettan Dalt*twelve* Separation Unit (GE Healthcare, USA). The gels were then fixed using gel fixative solution and stained with Coomasie dye. Finally, the gel was destained with destaining solution and imaged using Ettan DIGE Imager (GE Healthcare, USA). Three independent gels were run for each treatment (n = 3). Gel images were analyzed using PDQuest 2-D Analysis Software (Bio-Rad, USA) which performed background removal, normalization, and automatic matching of the detected protein spots. Protein spots with more than 2-fold differential expression that showed significant difference (p < 0.05) were selected and excised for mass spectrometry analysis.

### In-gel enzyme digestion

Protein spots excised from gels were first destained using 50% acetonitrile (ACN) in 50 mM ammonium bicarbonate and then incubated with 10mM DTT for 30 minutes at 60°C followed by incubation with 55 mM iodoacetamide for 20 minutes in the dark. The gel plugs were then washed with 50% ACN in 100 mM ammonium bicarbonate before incubation with 100% ACN for 15 min on a shaker and dried using SpeedVac. The dried gel plugs were subsequently incubated with 6 ng/μl trypsin in 50 mM ammonium bicarbonate overnight at 37°C, vortexed briefly and spun down before 50% and 100% ACN were added and shaken for 15 minutes each. The supernatant of each round of extraction was transferred into a fresh tube before the digested samples were completely dried using SpeedVac. The samples can be kept at -20°C until further use. Otherwise, extracted peptides were concentrated or desalted using ZipTip C18 microcolumns (Merck Millipore, Germany).

### MALDI-TOF/TOF mass spectrometry and database searching

Prior to MALDI-TOF/TOF analysis, 3 μl of each extracted peptide sample solution was mixed with 3 μl of alpha-cyano-4-hydroxycinnamic acid (Sigma-Aldrich, USA) matrix solution dissolved in 50% aqueous ACN containing 0.1% trifluoroacetic acid (Sigma-Aldrich, USA). A volume of 0.7 μl of each sample was applied onto a MALDI plate and was allowed to air dry at room temperature. Analysis was performed with ABSCIEX 4800 MALDI-TOF/TOF (AB SCIEX, USA) operated in the reflector for MALDI-TOF/TOF with fully automated mode using the 4800 Series Explorer software at an accelerating voltage of 20 kV. Calibration was performed using Mass Standards Kit for Calibration of AB SCIEX TOF/TOF™ Instruments (AB SCIEX, USA). Data collected from the MALDI-TOF/TOF were submitted to the SwissProt database using the MASCOT search algorithm (version 2.1.0, Matrix Science, London, UK). Typical search parameters for both search engines were defined as follows: trypsin digestion allowing up to two tryptic-mass cleavages, variable modifications of oxidation and carbamidomethyl, maximal mass tolerance of 0.1Da, precursor tolerance of 100 ppm, and taxonomy *Homo sapiens*. Protein scores greater than 55 were considered significant (p < 0.05). The protein with the highest number of peptides was considered as those corresponding to the spot if multiple proteins were identified in a single spot. The proteins identified were then compared with Uniprot KB/Swiss-Prot database and grouped according to the Eukaryotic Orthologous Group of Classifications (COGs).

### Data analysis

Results were expressed as the mean ± Standard Error Mean (SEM) of data obtained from three independent experiments. All data were analyzed using one way ANOVA, followed by Dunnett’s test for pairwise comparison. *P* < 0.05 was considered statistically significant for all tests.

## Results

### Determination of signaling pathways affected by *Phyllanthus*

**Figure 1:**
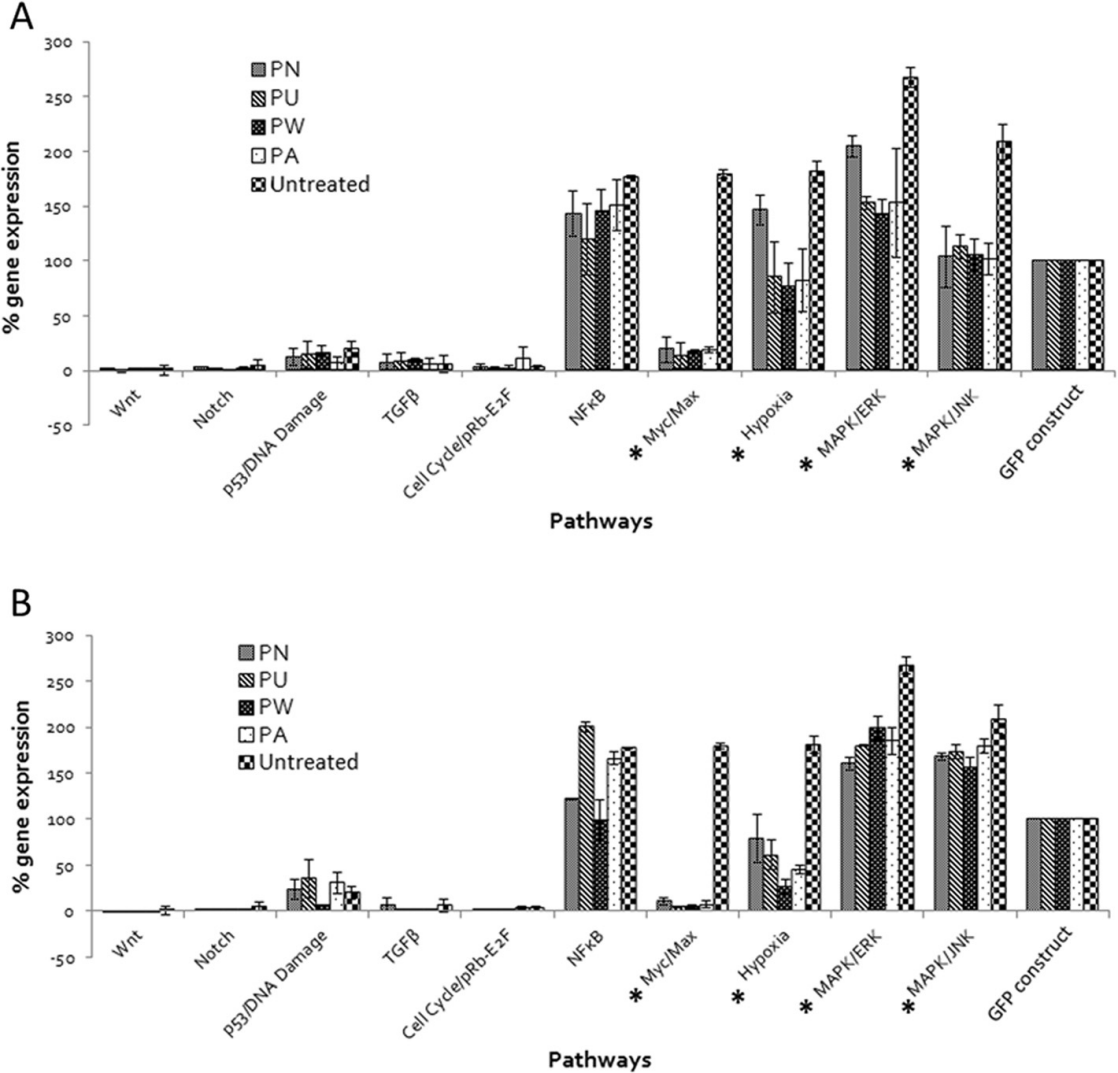
**Expression level of ten cellular signaling pathways in A549 cells treated with (a) aqueous *Phyllanthus *extracts and (b) methanolic *Phyllanthus *extracts.** Error bar indicates the standard error of the mean of three independent experiments. PN – *P. niruri*, PU – *P. urinaria*, PW – *P. watsonii*, PA – *P. amarus*. *P < 0.05 vs untreated-control.

The Cignal Finder Cancer 10-pathway reporter array was used to simultaneous screen 10 main cellular pathways that are targeted by *Phyllanthus* in its anticancer activities. The pathways included in this array include Wnt, Notch, p53/DNA damage, TGFβ, Cell cycle/pRb-E2F, NFκB, Myc/Max, Hypoxia, MAPK/ERK, and MAPK/JNK, with GFP construct plasmid DNA as the positive control for this array. As shown in Figure 1A (aqueous extracts-treated A549) and 1B (methanolic extracts-treated A549), the expression of GFP construct was consistent in both the aqueous and methanolic extracts-treated and untreated-control A549 cells, hence the results obtained were deemed valid. Upon treatment with various aqueous and methanolic extracts, most of these pathways’ expression [Myc/Max, Hypoxia, and MAPK (ERK and JNK)] decreased significantly (*p* < 0.05) except for NFκB which indicate *Phyllanthus* probably did not modulate this pathway. The aqueous *Phyllanthus* extracts showed better inhibitory activity on the expression of both MAP kinase pathways, while the methanolic extracts showed enhanced inhibition on the expression of Hypoxia and Myc/Max pathways. Among the four plant species, *P. watsonii* exhibited greatest suppression on Hypoxia (aqueous – 60% and methanolic – 86%), ERK (aqueous – 47% and methanolic – 27%), and JNK (aqueous – 50% and methanolic – 26%) pathways, followed by *P. urinaria*, *P. amarus*, and *P. niruri*.

**Figure 2:**
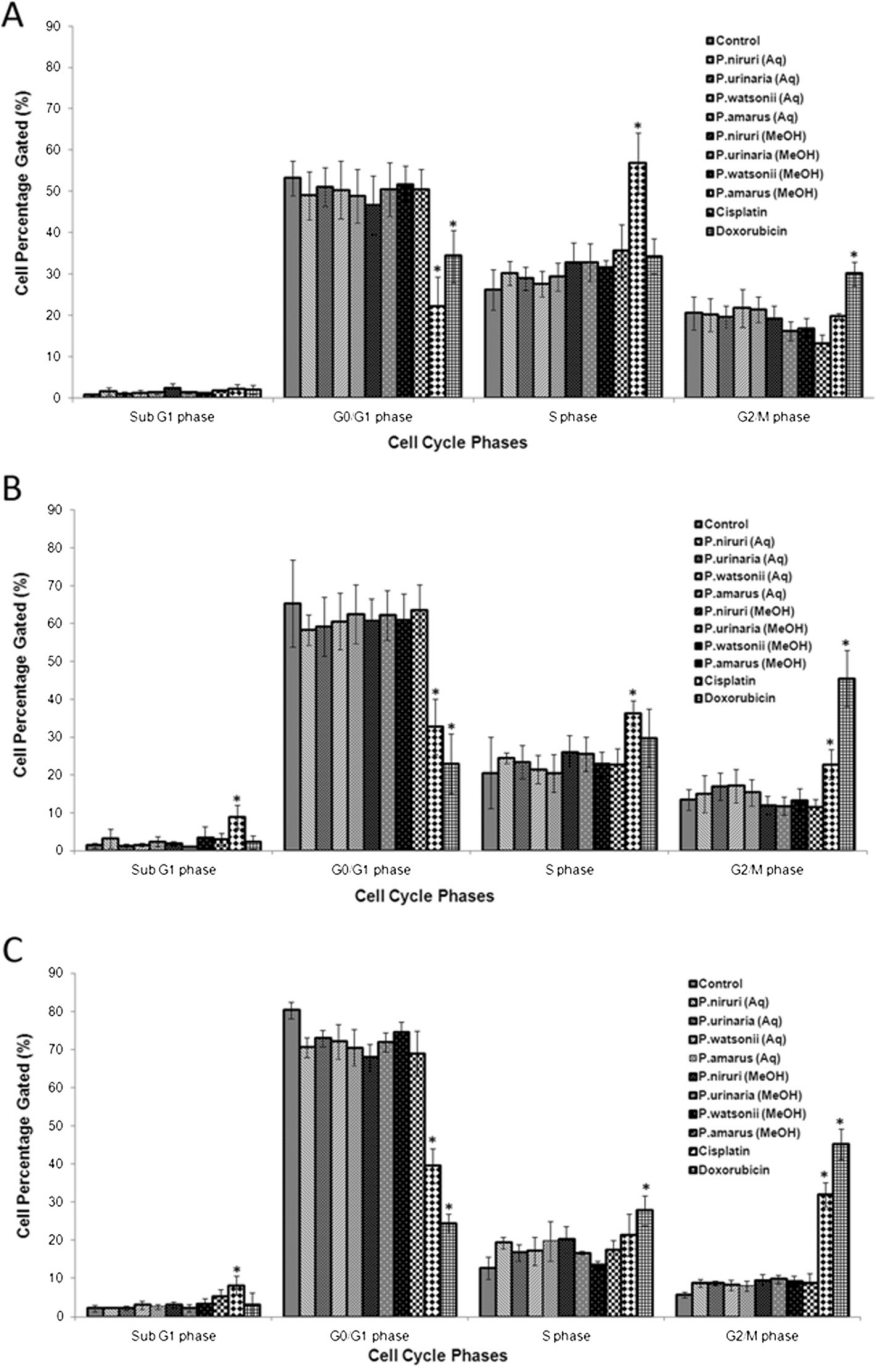
**Percentage of cell cycle phase distribution of A549 cells treated with both aqueous and methanolic *Phyllanthus *extracts and standard drugs at their IC**_**50 **_**(μg/ml) concentrations for (a) 24 hours, (b) 48 hours, and (c) 72 hours.** Error bar indicates the standard error of the mean of three independent experiments. Aq – Aqueous, MeOH - Methanolic, Control – Untreated cells. *P < 0.05 vs untreated-control.

From the flow cytomery-based cell cycle analysis, we did not observe cell cycle phase arrest in the A549 cells treated with *Phyllanthus* extracts (Figure 2) since the percentage of gated cells for each cell cycle phases (G_0_/G_1_, S, and G_2_/M) did not change significantly (*p* > 0.05) between the untreated and extracts-treated cells. Thus, this further explained the low expression level of cell cycle/pRb-E2F pathway in both the untreated and treated A549 cells. Nevertheless, the percentages decreased with a concurrent increase in the percentage of apoptotic cells (increase in Sub G_1_ phase) at increasing incubation time points (24, 48, and 72 hours). Conversely, there was an accumulation of cells at G_2_/M phase for A549 cells treated with Cisplatin and Doxorubicin.

### Modulation of intracellular signaling molecules expression by *Phyllanthus*

**Figure 3:**
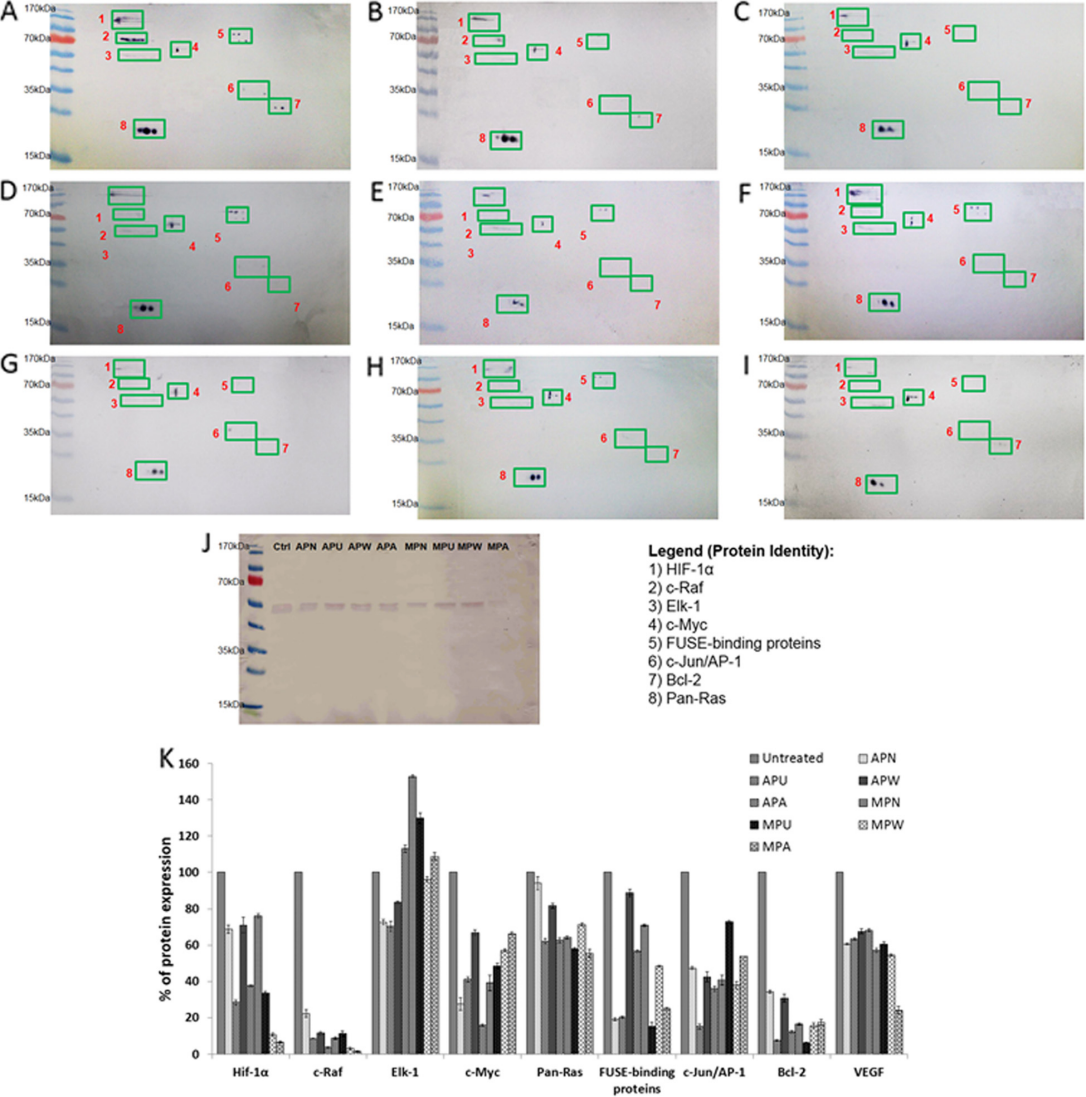
**Expression level of Pan-Ras, c-Raf, c-Jun/AP-1, Elk-1, c-Myc, HIF-1α, Bcl-2, and FUSE-binding proteins in (a) untreated A549 cells and cells treated with (b) aqueous *P. niruri, *** **(c) aqueous *P. urinaria, *** **(d) aqueous *P. watsonii, *** **(e) aqueous *P. amarus, *** **(f) methanolic *P. niruri, *** **(g) methanolic *P. urinaria, *** **(h) methanolic *P. watsonii, *** **and (i) methanolic *P. amarus*, (j) Expression level of VEGF in untreated-control and *Phyllanthus*-treated A549 cells, (k) Percentage of individual protein expression analysed using Image J software.**

In order to confirm the effects of *Phyllanthus* on MAPK and Hypoxia pathways, western blots were performed with available antibodies (Anti-pan-Ras, anti-c-Raf, anti-c-Myc, anti-Bcl-2, anti-Hif-1α, anti-c-Jun/AP-1, anti-p53, anti-Elk1, anti-JNK1/2, anti-VEGF, and anti-RSK) to determine the specific targets of *Phyllanthus*, whether it affects during the early or late stages of the signaling cascade. Figure 3A to I showed the blots for untreated A549 control as well as the A549 treated with various aqueous and methanolic extracts while Figure 3K depicts the expression level of each protein. In untreated A549, the proteins detected were Pan-Ras, c-Raf, c-Jun/AP-1, Elk-1, c-Myc, and HIF-1α. The presence of these proteins reflects the specific involvement of MAPK/ERK and Hypoxia pathways in regulating the A549 cells’ growth and survival. In addition, Bcl-2 protein was also detected in untreated A549 which explains its role as an antiapoptotic agent to ensure cell’s survival [27]. Besides that, FUSE-binding proteins were also detected although antibody specific to this protein was not included in this experiment. This might probably be attributed to its role for proper regulation of the c-Myc protooncogene [28], as it has a certain percentage of similarity with c-Myc protein and was therefore detected when c-Myc antibody was used. As expected, most of these proteins’ expression decreased when A549 was treated with various *Phyllanthus* extracts. Among the four species, *P. urinaria* showed better inhibition on those proteins, followed by *P. amarus*, *P. watsonii*, and *P. niruri* for both the aqueous and methanolic extracts. The expression of Pan-Ras and Elk-1 proteins were only mildly affected as compared to the other proteins and p53 expression was hardly seen in both the treated and untreated A549 cells via western blot technique, thus confirming the findings obtained from the previous cancer 10-pathway array which demonstrated low p53 expression in A549.

### Inhibition of matrix metalloproteinases (MMPs) expression by *Phyllanthus*

**Figure 4:**
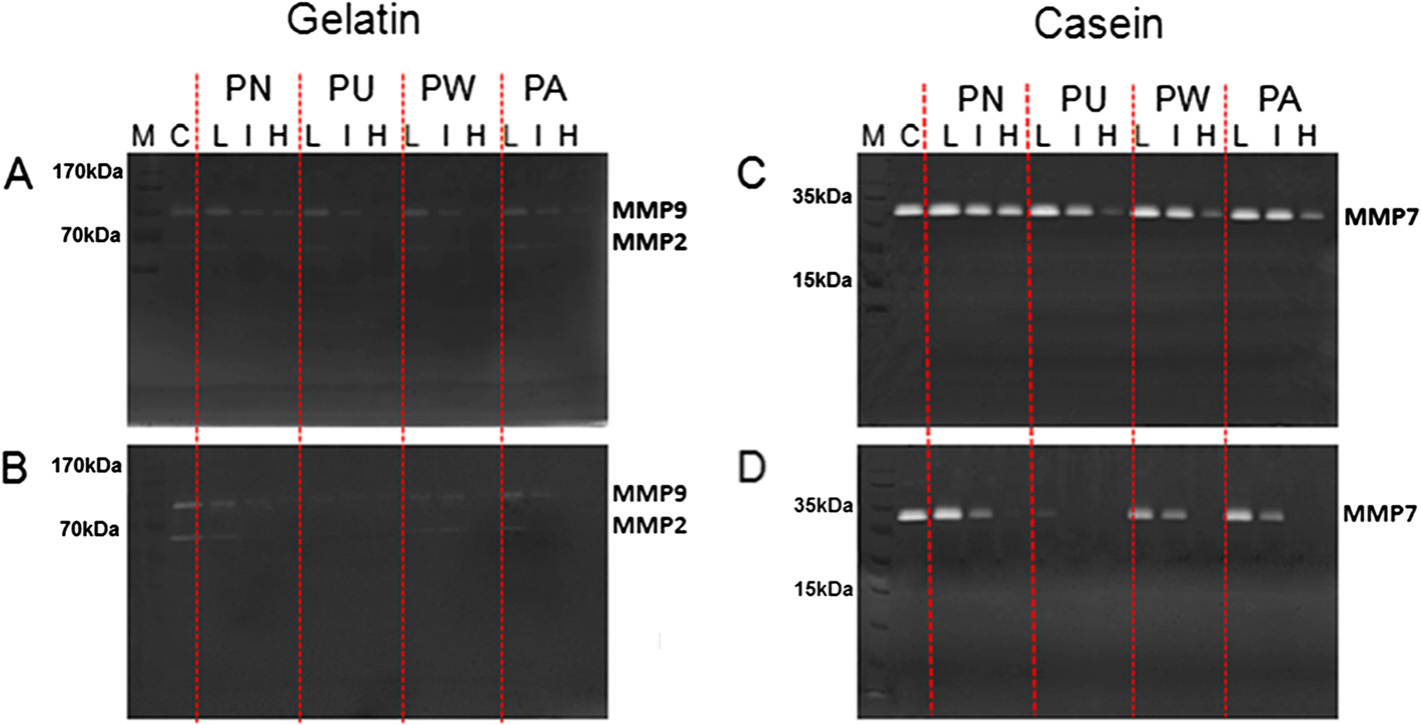
**Matrix metalloproteinases (MMPs) expression level in A549 cells treated with (a and c) aqueous *Phyllanthus *extracts and (b and d) methanolic *Phyllanthus *extracts.** M – protein marker, C – untreated control, L – treatment at 200 μg/ml and 20 μg/ml for aqueous and methanolic extracts respectively, I – treatment at their respective IC_50_ concentrations, H – treatment at 500 μg/ml and 50 μg/ml for aqueous and methanolic extracts respectively, PN – *P. niruri*, PU – *P. urinaria*, PW – *P. watsonii*, PA – *P. amarus*.

MMPs play an important role during tumor metastasis and angiogenesis since its expression level is often correlated with the tumor invasiveness [29]. Among the variety of MMPs, MMP2, MMP7, and MMP9 were more commonly associated with cancer metastasis as they have the ability to degrade collagen type IV which is the major component of basement membrane [23,25]. MMP7 probably plays a greater role in A549 metastasis since its expression was higher with brighter and clearer bands as compared to MMP2 and MMP9. Nonetheless, their expressions decreased in a dose-dependent manner as shown by the untreated-control and *Phyllanthus*-treated bands’ intensity in Figure 4. However, the bands for proMMPs with their respective MMPs were not well separated in the zymogram, hence leading to the inability to distinguish between the active and latent MMPs. Methanolic *Phyllanthus* extracts demonstrated greater inhibition on the MMPs’ expression than aqueous extracts, with *P. urinaria* showing the greatest inhibitory activity compared to the other *Phyllanthus* species.

### Inhibition of iNOS and VEGF expression by *Phyllanthus*

**Figure 5:**
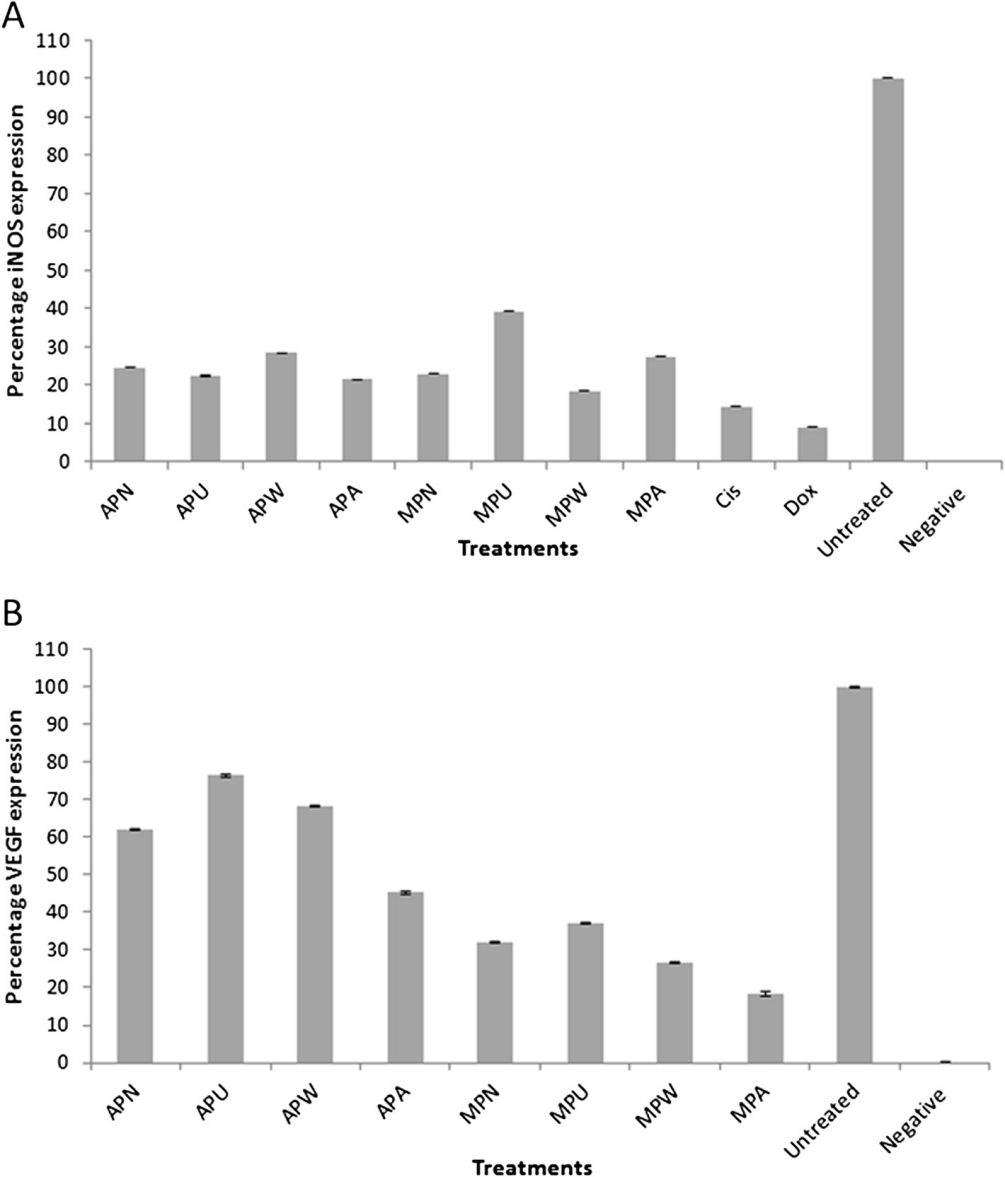
**Expression level of (a) iNOS and (b) VEGF in untreated and *Phyllanthus*-treated A549 cells.** APN - aqueous *P. niruri,* APU - aqueous *P. urinaria,* APW - aqueous *P. watsonii,* APA - aqueous *P. amarus,* MPN - methanolic *P. niruri,* MPU - methanolic *P. urinaria,* MPW - methanolic *P. watsonii,* MPA - methanolic *P. amarus*, CIS – Cisplatin, DOX – Doxorubicin. Error bar indicates the standard error of the mean of three independent experiments. *P* < 0.05 for all extracts-treated A549 compared to untreated-control A549.

Angiogenesis or blood vessel formation is the key process in the survival and metastasis of tumors in a hypoxic environment. During hypoxia, cytoplasmic HIF-1α subunit stabilization will lead to the activation of various genes including vascular endothelial growth factor (VEGF) and inducible nitric oxide synthases (iNOS) [30]. This was verified with the high expression of both VEGF and iNOS detected in the untreated-control A549 cells (Figure 5) using a cell-based ELISA assay. Upon treatment with various *Phyllanthus*, both the aqueous and methanolic extracts for all four species were observed to demonstrate inhibition on iNOS, whereby most of their expressions dropped markedly to approximately 20% except for methanolic *P. urinaria* which retained 40% of iNOS expression. Generally, *P. urinaria* (78% reduction) scored highest iNOS inhibition among aqueous extracts while *P. watsonii* (82% reduction) exhibited strongest activity among methanolic extracts. This suppression ability was comparable to Cisplatin and Doxorubicin with 15% and 10% iNOS expression that remained after treatment respectively.

On the other hand, methanolic *Phyllanthus* extracts showed better suppression of VEGF expression with 60 – 80% reduction compared to aqueous extracts which caused 20 – 50% reduction. In addition to this ELISA assay, expression of VEGF was also confirmed by western blot assay when VEGF expression dropped to 30 – 80% after A549 cells were treated with aqueous and methanolic *Phyllanthus* extracts (Figure 3J and K). Among the four species, methanolic *P. amarus* displayed better VEGF repression with only 20% VEGF expression. Meanwhile, *P. urinaria* exhibited slightly weaker capability to inhibit VEGF expression with 25% (aqueous) and 65% (methanolic) reduction respectively.

### Differentially expressed proteins in *Phyllanthus*-treated A549

**Figure 6:**
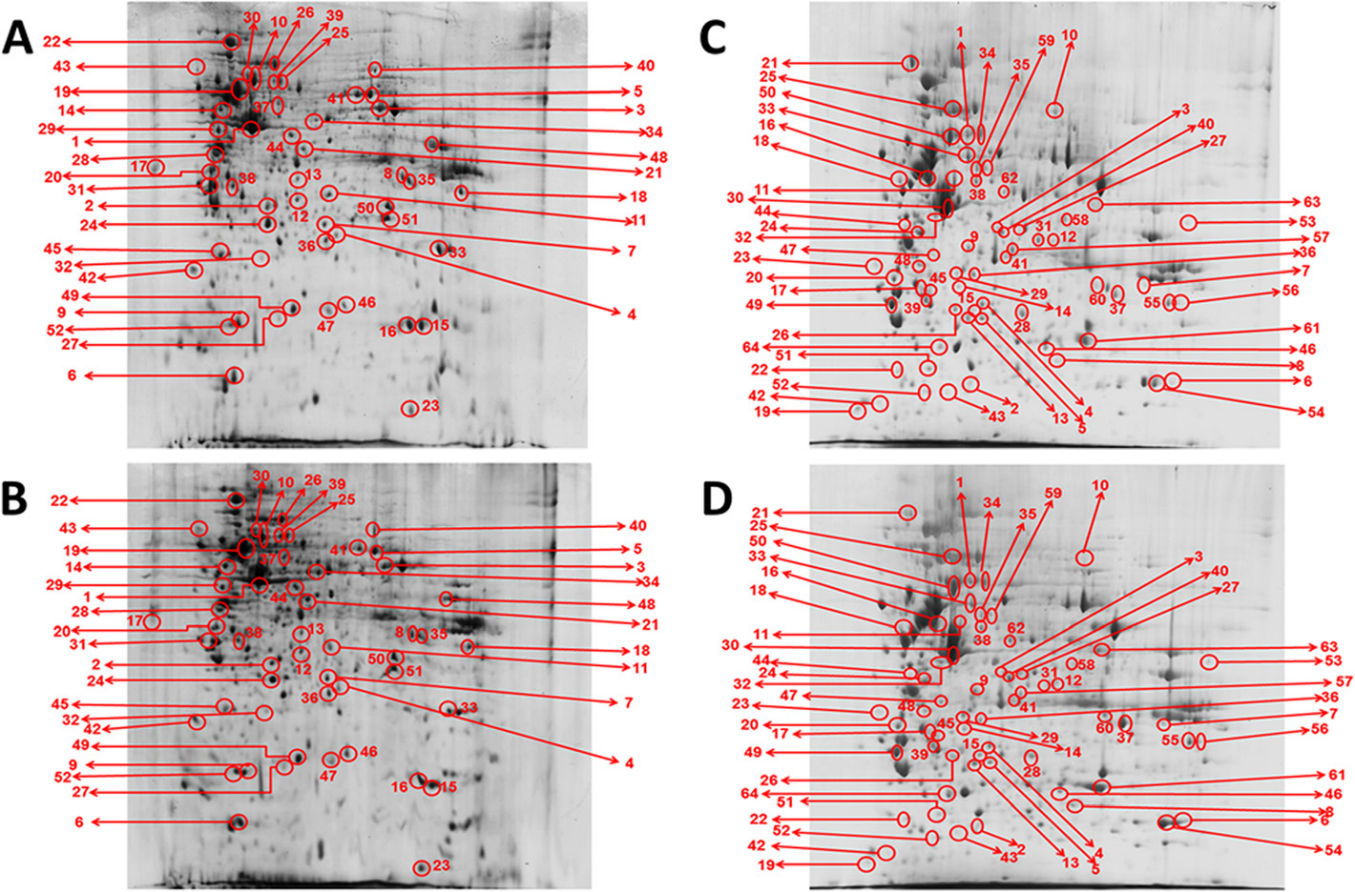
**Representative 2D-PAGE gels for (a and b) untreated-control A549 and its aqueous *P. watsonii*-treated A549, as well as (c and d) untreated-control A549 and its methanolic *P. watsonii*-treated A549.**

**Figure 7:**
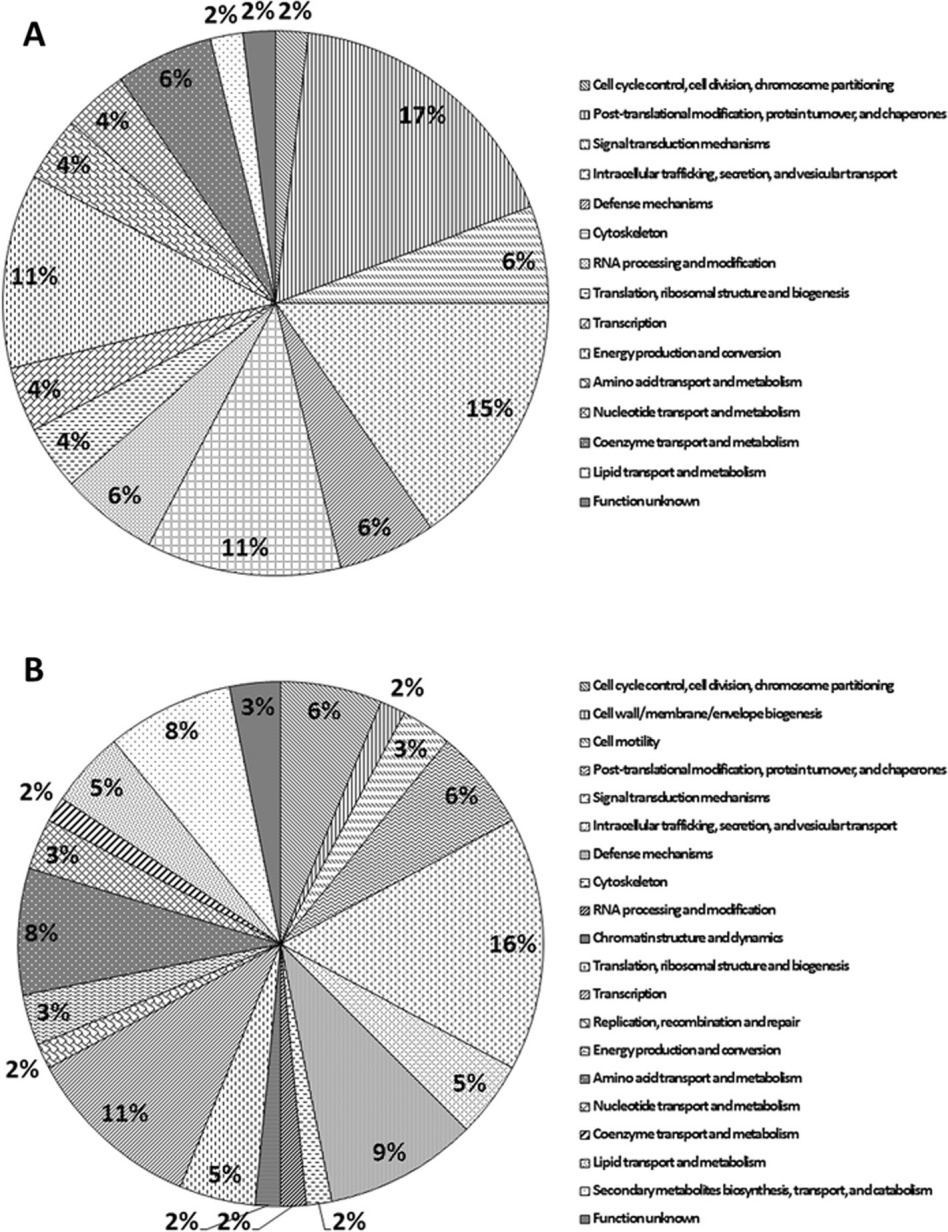
**Clusters of Orthologous Groups (COGs) classification of identified proteins in A549 cells treated with (a) aqueous *Phyllanthus *** **extracts and (b) methanolic *Phyllanthus *** **extracts.**

Figure 6 showed representative 2D-PAGE gels for untreated-control, aqueous *P. watsonii*-treated, and methanolic *P. watsonii*-treated A549 samples. Two-dimensional gel electrophoresis proteomic analysis picked out 68 and 79 protein spots differentially expressed in aqueous and methanolic extracts-treated groups respectively. Subsequent mass spectrometry analysis and database examination using MASCOT identified 52 protein spots significantly downregulated by aqueous *Phyllanthus* extracts as listed in Table 2. These protein spots were further categorized according to Clusters of Orthologous Groups (COGs) classification and majority of them fell into the category of post-translational modification, protein turnover, and chaperones, followed by intracellular trafficking, secretion, and vesicular transport, cytoskeleton, as well as energy production and conversion (Figure 7A). On the other hand, methanolic *Phyllanthus* extracts significantly suppressed 64 proteins as tabulated in Table 3. Most of these proteins fell into the category of signal transduction mechanisms, transcription, defense mechanisms, amino acid transport and metabolism, as well as secondary metabolites biosynthesis, transport, and catabolism (Figure 7B). The negative (-) symbol in both Tables 2 and 3 signified suppression of the proteins and a value of 1.00 indicates the complete absence of this protein in the *Phyllanthus*-treated sample as compared to the untreated-control sample and the degree of suppression reduces as the value increases.

**Table 2:** **Identified proteins of A549 downregulated (-) in the presence of aqueous *Phyllanthus *** **extracts**

**Spot ID**	**Possible proteins**	**Fold change**				**UNIPROT KB/SWISS-PROT Acc. Number**	**Database**
		**APN**	**APU**	**APW**	**APA**		
1	Actin, cytoplasmic 1	-0.81	-0.88	-0.95	-0.84	P60709	MASCOT;UNIPROT KB/SWISS-PROT;COGS
2	Nicotinamide N-methyltransferase	-0.50	N/A	-0.28	-0.10	P40261	MASCOT;UNIPROT KB/SWISS-PROT;COGS
3	Alpha-Enolase	-0.62	-0.57	-0.48	-0.58	P06733	MASCOT;UNIPROT KB/SWISS-PROT;COGS
4	Proteasome subunit beta type 3	-1.00	-0.20	N/A	-1.00	P49720	MASCOT;UNIPROT KB/SWISS-PROT;COGS
5	Retinal dehydrogenase 1	-0.23	N/A	-0.13	N/A	P00352	MASCOT;UNIPROT KB/SWISS-PROT;COGS
6	Galectin-1	-1.00	-0.13	-0.57	-0.38	P09382	MASCOT;UNIPROT KB/SWISS-PROT;COGS
7	Heat-shock protein beta-1	-0.12	-0.22	-0.32	-0.10	P04792	MASCOT;UNIPROT KB/SWISS-PROT;COGS
8	Glyceraldehyde-3-phosphate dehydrogenase	-0.30	-0.93	-0.99	-0.91	P04406	MASCOT;UNIPROT KB/SWISS-PROT;COGS
9	Eukaryotic translation initiation factor 5A-1	-0.32	-0.39	-0.27	-0.80	P63241	MASCOT;UNIPROT KB/SWISS-PROT;COGS
10	60 kDa heat shock protein, mitochondrial precursor	-0.46	-0.82	-0.90	-0.75	P10809	MASCOT;UNIPROT KB/SWISS-PROT;COGS
11	Glutathione transferase omega-1	N/A	-0.45	N/A	-0.22	P78417	MASCOT;UNIPROT KB/SWISS-PROT;COGS
12	Proteasome activator complex subunit 1	-0.19	-0.18	N/A	-1.00	Q06323	MASCOT;UNIPROT KB/SWISS-PROT;COGS
13	Annexin A4	-0.29	-0.29	-0.21	-0.65	P09525	MASCOT;UNIPROT KB/SWISS-PROT;COGS
14	Vimentin	-0.91	-0.82	-0.24	-0.79	P08670	MASCOT;UNIPROT KB/SWISS-PROT;COGS
15	Peptidyl-prolyl cis-trans isomerase A	-0.13	-0.36	-1.00	-0.42	P62937	MASCOT;UNIPROT KB/SWISS-PROT;COGS
16	Peptidyl-prolyl cis-trans isomerase A	-1.00	-1.00	-0.32	N/A	P62937	MASCOT;UNIPROT KB/SWISS-PROT;COGS
17	Glyceraldehyde-3-phosphate dehydrogenase	-1.00	-0.50	-1.00	-0.71	P04406	MASCOT;UNIPROT KB/SWISS-PROT;COGS
18	Voltage-dependent anion-selective channel protein 1	-0.37	-0.19	-0.20	N/A	P21796	MASCOT;UNIPROT KB/SWISS-PROT;COGS
19	Tubulin alpha-8 chain	-0.80	-0.37	N/A	-0.80	Q9NY65	MASCOT;UNIPROT KB/SWISS-PROT;COGS
20	Protein FAM24B precursor	-0.51	-0.54	-0.66	-0.88	Q8N5W8	MASCOT;UNIPROT KB/SWISS-PROT;COGS
21	Interferon alpha-6 precursor	-0.41	N/A	-0.38	-0.75	P05013	MASCOT;UNIPROT KB/SWISS-PROT;COGS
22	ATP-dependent DNA helicase Q5	-1.00	-1.00	-0.93	-0.79	O94762	MASCOT;UNIPROT KB/SWISS-PROT;COGS
23	Sorting nexin-3	-0.25	-1.00	-1.00	-1.00	O60493	MASCOT;UNIPROT KB/SWISS-PROT;COGS
24	Gap junction beta-5 protein	-0.36	-0.16	-0.32	-0.41	O95377	MASCOT;UNIPROT KB/SWISS-PROT;COGS
25	40S ribosomal protein S19	-0.30	-0.30	-0.15	-0.38	P39019	MASCOT;UNIPROT KB/SWISS-PROT;COGS
26	Stress-70 protein, mitochondrial precursor	-0.19	-0.22	N/A	N/A	P38646	MASCOT;UNIPROT KB/SWISS-PROT;COGS
27	Actin-related protein 2/3 complex subunit 5	-1.00	-1.00	-1.00	-1.00	O15511	MASCOT;UNIPROT KB/SWISS-PROT;COGS
28	Sorting nexin-3	-0.36	-0.35	-0.37	-1.00	O60493	MASCOT;UNIPROT KB/SWISS-PROT;COGS
29	Complement receptor type 1 precursor	-0.19	N/A	-0.41	-1.00	P17927	MASCOT;UNIPROT KB/SWISS-PROT;COGS
30	40S ribosomal protein S24	-1.00	-1.00	-1.00	-1.00	P62847	MASCOT;UNIPROT KB/SWISS-PROT;COGS
31	Corticotropin-lipotropin precursor	-0.73	-0.88	-1.00	-1.00	P01189	MASCOT;UNIPROT KB/SWISS-PROT;COGS
32	Inosine triphosphate pyrophosphatase	-1.00	-0.67	-1.00	-1.00	Q9BY32	MASCOT;UNIPROT KB/SWISS-PROT;COGS
33	Peroxiredoxin-1	-0.08	-0.56	-1.00	-0.33	Q06830	MASCOT;UNIPROT KB/SWISS-PROT;COGS
34	Protein memo	-0.95	-0.88	-0.56	-0.47	Q9Y316	MASCOT;UNIPROT KB/SWISS-PROT;COGS
35	Voltage-dependent anion-selective channel protein 2	-1.00	-0.31	-0.46	-0.21	P45880	MASCOT;UNIPROT KB/SWISS-PROT;COGS
36	Thioredoxin-dependent peroxide reductase, mitochondrial precursor	-0.28	-0.19	-0.27	N/A	P30048	MASCOT;UNIPROT KB/SWISS-PROT;COGS
37	RuvB-like 2	-0.24	N/A	-0.43	-0.11	Q9Y230	MASCOT;UNIPROT KB/SWISS-PROT;COGS
38	EF-hand domain-containing protein 2	-0.36	-0.17	-0.37	-0.29	Q5JST6	MASCOT;UNIPROT KB/SWISS-PROT;COGS
39	T-complex protein 1 subunit epsilon	-0.89	N/A	-0.20	-0.97	P48643	MASCOT;UNIPROT KB/SWISS-PROT;COGS
40	Stress-induced-phosphoprotein 1	-0.54	-0.62	-0.44	-0.21	P31948	MASCOT;UNIPROT KB/SWISS-PROT;COGS
41	Retinal dehydrogenase 1	-0.33	-0.79	-0.58	-0.61	P00352	MASCOT;UNIPROT KB/SWISS-PROT;COGS
42	Prostaglandin E synthase 3	-0.26	-0.36	-0.97	-0.16	Q15185	MASCOT;UNIPROT KB/SWISS-PROT;COGS
43	Trypsin-1 precursor	-0.37	-1.00	-0.28	-0.35	P07477	MASCOT;UNIPROT KB/SWISS-PROT;COGS
44	Serpin B9	-0.51	-0.14	-0.21	-0.51	P50453	MASCOT;UNIPROT KB/SWISS-PROT;COGS
45	N-acylneuraminate cytidylyltransferase	-0.64	-0.57	-0.50	-0.28	Q8NFW8	MASCOT;UNIPROT KB/SWISS-PROT;COGS
46	Cofilin-1	-0.76	N/A	-0.85	-0.38	P23528	MASCOT;UNIPROT KB/SWISS-PROT;COGS
47	ADP-ribosylation factor 1	-1.00	-0.21	-0.15	-1.00	P84077	MASCOT;UNIPROT KB/SWISS-PROT;COGS
48	Fructose-bisphosphate aldolase A	-1.00	-1.00	-1.00	-1.00	P04075	MASCOT;UNIPROT KB/SWISS-PROT;COGS
49	Sorting nexin-3	-1.00	-1.00	-1.00	-1.00	O60493	MASCOT;UNIPROT KB/SWISS-PROT;COGS
50	Phosphoglycerate mutase 1	-1.00	-1.00	-1.00	-1.00	P18669	MASCOT;UNIPROT KB/SWISS-PROT;COGS
51	Triosephosphate isomerase	-1.00	-1.00	-1.00	-1.00	P60174	MASCOT;UNIPROT KB/SWISS-PROT;COGS
52	Eukaryotic translation initiation factor 5A-1	-1.00	-1.00	-1.00	-1.00	P63241	MASCOT;UNIPROT KB/SWISS-PROT;COGS

The negative (-) symbol signified suppression of the proteins and a value of 1.00 indicates the complete absence of this protein in the aqueous *Phyllanthus*-treated sample as compared to the untreated-control sample and the degree of suppression reduces as the value increases. APN – Aqueous *P. niruri*, APU – Aqueous *P. urinaria*, APW – Aqueous *P. watsonii*, APA – Aqueous *P. amarus,* N/A – Not Affected.

**Table 3:** **Identified proteins of A549 downregulated (-) in the presence of methanolic *Phyllanthus *** **extracts**

**Spot ID**	**Possible proteins**	**Fold change**				**UNIPROT KB/SWISS-PROT Acc. Number**	**Database**
		**MPN**	**MPU**	**MPW**	**MPA**		
1	Protein S100-A8	-0.47	-0.10	-0.48	-0.55	P05109	MASCOT;UNIPROT KB/SWISS-PROT;COGS
2	Transmembrane protein 35	-0.82	-0.78	-1.00	-0.50	Q53FP2	MASCOT;UNIPROT KB/SWISS-PROT;COGS
3	Adenylate kinase isoenzyme 6	-0.32	-0.62	-0.81	-0.73	Q9Y3D8	MASCOT;UNIPROT KB/SWISS-PROT;COGS
4	Peroxiredoxin-1	-0.78	-1.00	-1.00	-1.00	Q06830	MASCOT;UNIPROT KB/SWISS-PROT;COGS
5	Prolactin-releasing peptide precursor	-0.59	-0.29	-0.23	-0.39	P81277	MASCOT;UNIPROT KB/SWISS-PROT;COGS
6	Putative protein SSX6	-0.46	-0.96	-1.00	-0.75	Q7RTT6	MASCOT;UNIPROT KB/SWISS-PROT;COGS
7	Glutathione synthetase	-0.38	-0.79	-1.00	-0.51	P48637	MASCOT;UNIPROT KB/SWISS-PROT;COGS
8	Nucleoside diphosphate-linked moiety X motif 16	-0.61	-0.63	-0.98	-0.94	Q3MHX9	MASCOT;UNIPROT KB/SWISS-PROT;COGS
9	E3 ubiquitin-protein ligase ZNRF1	N/A	N/A	-0.47	-0.36	Q8ND25	MASCOT;UNIPROT KB/SWISS-PROT;COGS
10	DNA-directed RNA polymerase II 16 kDa polypeptide	-0.46	-0.59	-0.43	-0.93	Q9VEA5	MASCOT;UNIPROT KB/SWISS-PROT;COGS
11	39S ribosomal protein L40, mitochondrial precursor	-1.00	-1.00	-1.00	-1.00	Q9NQ50	MASCOT;UNIPROT KB/SWISS-PROT;COGS
12	Zinc finger protein 174	-1.00	-1.00	-1.00	-1.00	Q15697	MASCOT;UNIPROT KB/SWISS-PROT;COGS
13	Probable G-protein coupled receptor 179 precursor	-0.21	-0.15	-1.00	N/A	Q6PRD1	MASCOT;UNIPROT KB/SWISS-PROT;COGS
14	Histatin-1 precursor	-0.24	-0.66	-0.78	-0.27	P15515	MASCOT;UNIPROT KB/SWISS-PROT;COGS
15	U6 snRNA-associated Sm-like protein LSm5	-0.33	-0.57	-0.19	-0.54	Q9Y4Y9	MASCOT;UNIPROT KB/SWISS-PROT;COGS
16	Bcl-2-like protein 11	-0.25	-1.00	-1.00	-0.70	O43521	MASCOT;UNIPROT KB/SWISS-PROT;COGS
17	Contactin-2 precursor	-0.29	-0.60	-0.42	-0.39	Q02246	MASCOT;UNIPROT KB/SWISS-PROT;COGS
18	Bis(5′-adenosyl)-triphosphatase	-1.00	-1.00	N/A	-1.00	P49789	MASCOT;UNIPROT KB/SWISS-PROT;COGS
19	Trypsin-1 precursor	-1.00	-0.98	-1.00	-1.00	P07477	MASCOT;UNIPROT KB/SWISS-PROT;COGS
20	Proliferating cell nuclear antigen	-1.00	-1.00	-1.00	-1.00	P12004	MASCOT;UNIPROT KB/SWISS-PROT;COGS
21	Endoplasmin precursor	N/A	-1.00	-1.00	-0.37	P14625	MASCOT;UNIPROT KB/SWISS-PROT;COGS
22	Eukaryotic translation initiation factor 3 subunit 12	-1.00	-1.00	-1.00	-1.00	Q9UBQ5	MASCOT;UNIPROT KB/SWISS-PROT;COGS
23	Serine/threonine-protein kinase 6	-1.00	-0.96	-0.19	-1.00	O14965	MASCOT;UNIPROT KB/SWISS-PROT;COGS
24	Phenylalanyl-tRNA synthetase beta chain	-1.00	-1.00	-1.00	-1.00	Q9NSD9	MASCOT;UNIPROT KB/SWISS-PROT;COGS
25	Neutrophil defensin 1 precursor	-0.78	-0.49	-0.20	-0.88	P59665	MASCOT;UNIPROT KB/SWISS-PROT;COGS
26	Proto-oncogene protein Wnt-3 precursor	-0.58	N/A	-0.34	-0.55	P56703	MASCOT;UNIPROT KB/SWISS-PROT;COGS
27	Putative Ras-related protein Rab-42	-1.00	N/A	-0.97	-0.80	Q8N4Z0	MASCOT;UNIPROT KB/SWISS-PROT;COGS
28	UPF0404 protein C11orf59	-0.19	-0.36	N/A	-0.36	Q6IAA8	MASCOT;UNIPROT KB/SWISS-PROT;COGS
29	Neutrophil defensin 1 precursor	-0.31	-0.25	-1.00	-0.87	P59665	MASCOT;UNIPROT KB/SWISS-PROT;COGS
30	Agouti-signaling protein precursor	-0.33	-0.67	-0.67	N/A	P42127	MASCOT;UNIPROT KB/SWISS-PROT;COGS
31	DNA-directed RNA polymerase II 16 kDa polypeptide	-0.42	-0.48	-0.48	-0.45	Q9VEA5	MASCOT;UNIPROT KB/SWISS-PROT;COGS
32	Beta-defensin 107A precursor	-0.21	N/A	-0.62	-0.61	Q8IZN7	MASCOT;UNIPROT KB/SWISS-PROT;COGS
33	Transcription elongation factor B polypeptide 1	-0.58	-0.59	-0.56	-0.79	Q15369	MASCOT;UNIPROT KB/SWISS-PROT;COGS
34	Metallothionein-1M	-0.55	-0.52	-0.48	-0.48	Q8N339	MASCOT;UNIPROT KB/SWISS-PROT;COGS
35	GTPase HRas precursor	-0.60	-0.66	-0.59	-0.61	P01112	MASCOT;UNIPROT KB/SWISS-PROT;COGS
36	Neuromedin-B precursor	-0.24	-0.32	-0.23	-0.34	P08949	MASCOT;UNIPROT KB/SWISS-PROT;COGS
37	Protein-tyrosine sulfotransferase 2	-0.36	N/A	N/A	-0.33	O60704	MASCOT;UNIPROT KB/SWISS-PROT;COGS
38	Apolipoprotein A-II precursor	-0.23	-0.23	-0.13	-0.37	P02652	MASCOT;UNIPROT KB/SWISS-PROT;COGS
39	ADP-ribosylation factor-like protein 6	-0.58	-0.52	-1.00	-0.29	Q9H0F7	MASCOT;UNIPROT KB/SWISS-PROT;COGS
40	Alpha-enolase	-0.27	-0.51	N/A	-0.56	P06733	MASCOT;UNIPROT KB/SWISS-PROT;COGS
41	Serine/threonine-protein phosphatase PP1-beta catalytic subunit	-1.00	-1.00	-0.44	-0.71	P62140	MASCOT;UNIPROT KB/SWISS-PROT;COGS
42	N-acylneuraminate cytidylyltransferase	-1.00	-1.00	-1.00	-1.00	Q8NFW8	MASCOT;UNIPROT KB/SWISS-PROT;COGS
43	Inosine triphosphate pyrophosphatase	-1.00	-1.00	-1.00	-1.00	Q9BY32	MASCOT;UNIPROT KB/SWISS-PROT;COGS
44	Alkyldihydroxyacetonephosphate synthase	-1.00	-0.55	-1.00	-1.00	O00116	MASCOT;UNIPROT KB/SWISS-PROT;COGS
45	Microsomal signal peptidase 18 kDa subunit	-1.00	-0.57	-1.00	-1.00	P67812	MASCOT;UNIPROT KB/SWISS-PROT;COGS
46	Tumor suppressor candidate 2	-0.89	-0.75	-0.67	-0.21	O75896	MASCOT;UNIPROT KB/SWISS-PROT;COGS
47	Metallothionein-2	-0.71	-0.17	-0.14	-0.53	P02795	MASCOT;UNIPROT KB/SWISS-PROT;COGS
48	Protein FAM3B precursor	-0.57	-0.48	-0.39	-0.48	P58499	MASCOT;UNIPROT KB/SWISS-PROT;COGS
49	Metallothionein-1H	-0.52	-0.48	N/A	-0.29	P80294	MASCOT;UNIPROT KB/SWISS-PROT;COGS
50	Metallothionein-2	-0.34	-0.59	-0.49	-1.00	P02795	MASCOT;UNIPROT KB/SWISS-PROT;COGS
51	Protein BEX5	-0.48	-0.33	-0.30	-0.48	Q5H9J7	MASCOT;UNIPROT KB/SWISS-PROT;COGS
52	DNA-directed RNA polymerase II 16 kDa polypeptide	-1.00	-1.00	-1.00	-1.00	Q9VEA5	MASCOT;UNIPROT KB/SWISS-PROT;COGS
53	Cytochrome c oxidase polypeptide VIa-liver, mitochondrial precursor	-0.93	-0.24	-1.00	-0.14	P12074	MASCOT;UNIPROT KB/SWISS-PROT;COGS
54	Putative protein 15E1.2	N/A	-0.94	-1.00	-0.30	-	MASCOT;COGS
55	Voltage-dependent anion-selective channel protein 1	-0.15	-0.28	-0.35	-0.27	P21796	MASCOT;UNIPROT KB/SWISS-PROT;COGS
56	Voltage-dependent anion-selective channel protein 1	-0.47	-0.13	-1.32	N/A	P21796	MASCOT;UNIPROT KB/SWISS-PROT;COGS
57	Serine/threonine-protein phosphatase PP1-alpha catalytic subunit	-0.64	-0.76	-0.43	-0.32	P62136	MASCOT;UNIPROT KB/SWISS-PROT;COGS
58	Acetyl-CoA acetyltransferase, cytosolic	-0.95	-1.00	-0.17	-1.00	Q9BWD1	MASCOT;UNIPROT KB/SWISS-PROT;COGS
59	Glutathione synthetase	-0.50	-0.45	-0.38	-0.61	P48637	MASCOT;UNIPROT KB/SWISS-PROT;COGS
60	Actin-related protein 10	-0.94	-0.70	-0.13	-0.73	Q9NZ32	MASCOT;UNIPROT KB/SWISS-PROT;COGS
61	Enteropeptidase precursor	-0.63	-0.19	-1.00	-0.70	P98073	MASCOT;UNIPROT KB/SWISS-PROT;COGS
62	Metallothionein-1L	-0.86	-0.79	-0.96	-0.44	Q93083	MASCOT;UNIPROT KB/SWISS-PROT;COGS
63	Guanine nucleotide-binding protein G(I)/G(S)/G(O) gamma-5-like subunit	-1.00	-1.00	-1.00	-1.00	P63218	MASCOT;UNIPROT KB/SWISS-PROT;COGS
64	Small inducible cytokine B14 precursor	-1.00	-1.00	-1.00	-1.00	O95715	MASCOT;UNIPROT KB/SWISS-PROT;COGS

The negative (-) symbol signified suppression of the proteins and a value of 1.00 indicates the complete absence of this protein in the methanolic *Phyllanthus*-treated sample as compared to the untreated-control sample and the degree of suppression reduces as the value increases. MPN – Methanolic *P. niruri*, MPU – Methanolic *P. urinaria*, MPW – Methanolic *P. watsonii*, MPA – Methanolic *P. amarus,* N/A – Not Affected.

## Discussion

Numerous reports describe poor therapeutic efficacy and prognostic survival for lung malignancy which is largely due to its capability to invade, metastasize, as well as to induce angiogenesis. Therefore, it is crucial to develop novel antimetastatic drugs with low toxicity and high efficacy [17,29]. In our previous study, aqueous and methanolic extracts of *Phyllanthus* was shown to possess the potential to inhibit growth of A549 in a time- and dose-dependent manner with minimal toxicity to the normal lung epithelial (NL20) cells. Besides that, *Phyllanthus* suppressed the invasion, migration, and reattachment of A549 in a dose-dependent manner and was capable of inducing apoptosis in conjunction with its antimetastastic action [17]. However, the underlying mechanisms that confer their antimetastatic and apoptosis-inducing abilities were uncertain.

In the current study, numerous differentially expressed proteins were being identified in A549 cells in response to *Phyllanthus* treatments using 2DE-based proteomic approach. Both aqueous and methanolic *Phyllanthus* extracts modulate expression of different set of proteins [17]. Among the four *Phyllanthus* species, *P. urinaria* generally demonstrated the greatest activity on A549, closely followed by *P. watsonii*, *P. amarus*, and *P. niruri*. This could be delineated by the higher number of polyphenol compounds present in both the aqueous (9 out of 10 polyphenols) and methanolic (3 out of 4 polyphenols) *P. urinaria* extracts [17,20], hence having a higher capability to cause antimetastatic activities on A549. Some of those proteins were represented by more than one spot, which represents different splicing forms of the same protein as a result of post-translational modification [14].

Therefore, protein turnover, chaperones, and post-translational modification are indeed crucial to produce many variants of the common amino acid that possess distinctive structures and functions essential for tumor growth [31,32]. In order to ensure uninterrupted cell growth, continuous protein synthesis is necessary. Therefore, 40S ribosomal protein is normally upregulated in the tumor cell as it is an essential component of the higher eukaryotic ribosome that is necessary for proper protein translational function [33]. Eukaryotic translation initiation factor 3 subunit 12 is another important component as it binds to poly(A)-binding protein to initiate translation process [34]. Protein folding is the subsequent critical process upon successful protein translation to ensure formation of functional proteins and this step usually requires the presence of chaperone proteins. One of these proteins is Hsp60 whose primary role is to guide the folding of mitochondrial proteins while facilitating proteolytic degradation of denatured or misfolded proteins in an ATP-dependent manner [35]. Other chaperones include peptidyl-prolyl cis-trans isomerase A and T-complex protein 1 subunit epsilon which also assists the proper folding of proteins [36,37].

Besides facilitating protein folding, chaperones also guide the proper assembly of other proteins for them to carry out their activities. An important example is stress-induced phosphoprotein 1 that facilitates association of molecular chaperones Hsp70 and Hsp 90 which have been implicated in MMP2 activity that lead to increased invasiveness [38]. Downregulation of these proteins by *Phyllanthus* is therefore one of the key processes for A549 growth and metastasis inhibition by reducing functional protein synthesis and suppression of MMP expression. In addition to MMP2, suppression of other MMPs in response to *Phyllanthus* treatment was also observed including MMP7 and MMP9. Tumor invasion and metastasis depends largely on the integrity of the basement membrane which is frequently destroyed by a number of proteolytic enzymes such as MMPs in order to access the vasculature to develop distant metastases [22]. Among the MMPs family, MMP2 and MMP9 were more commonly associated with cancer invasion and metastasis since they had been known to be able to degrade type IV collagen-rich basement membrane of vessel wall [23]. MMP7 (28 kDa) is another member of the MMP family with broad substrate specificity against ECM components such as elastin, type IV collagen, fibronectin, vitronectin, aggrecan, and proteoglycans [25]. Reduced expression of these MMP enzymes explains the decreased aggressiveness of A549 cells’ invasion upon treatment with *Phyllanthus*.

A constant activation of various growth-promoting signaling pathways is also required to ensure continuous cell growth and survival. This activation often involves numerous proteins which require some form of modifications such as phosphorylation for their biological role. Thus, post-translational modification process that alters the proteins properties is important [39,40]. The first step for pathway activation often requires the presence of receptor molecules on the cell surface for ligand binding. So, guanine nucleotide-binding protein, putative Ras-related protein Rab-42, and GTPase HRas precursor protein which can be broadly grouped as G-proteins, as well as probable G-protein coupled receptor 179 precursor expressions are usually elevated in the tumor cells to accommodate the large amount of extracellular signals to be transduced into the cells [41]. Both G-protein coupled receptor and G-proteins form the G-protein mediated signaling cascade whereby binding of ligands to receptors will lead to activation of G-proteins by promoting GDP/GTP exchange, which in turn regulates many effector molecules such as proteins kinases [41]. Therefore, the subsequent upregulated protein in the tumor cell is serine/threonine-protein kinase 6 which is a family of kinases including MAP kinase [42], which functions to turn on the downstream kinases via serine/threonine phosphorylation [43]. Suppression of these cell signaling proteins by *Phyllanthus* could therefore decelerate or stop the constitutive activation of the growth-promoting pathways such as MAP kinase and hypoxia.

ERK1/2 pathway is one of the MAP kinase subgroup which was frequently found to be inhibited in A549 to repress cells’ continuous growth and metastasis. In a study by Shih et al. [21], α-tomatine found in tomatoes was shown to be capable of inactivating extracellular signal-regulated kinase 1 and 2 (ERK1/2) pathway to inhibit metastasis occurrence in A549 cell line. Similarly, A549 metastasis was also inhibited by Silibinin isolated from *Silybum marianum* which suppresses ERK1/2 pathway that led to a reduced expression of MMP2 and u-PA concomitantly with a significant inhibition on cell invasion [22]. Indeed, our study also showed that *Phyllanthus* inhibit A549 metastasis via targeting specifically ERK1/2 pathway. ERK1/2 module is often thought as a linear pathway since ERK is the effector of an evolutionarily conserved signaling component that is triggered exclusively by the Raf serine/threonine kinases [44,45]. Therefore, *Phyllanthus* might possibly downregulate Raf protein at the early stage of the pathway, resulting in the subsequent suppression of other proteins’ expression down the pathway. As a result, various biological activities controlled by ERK1/2 pathway to increase cell growth and malignancy are repressed, including regulation of transcriptional, cell cycle, apoptosis, and metastasis [46].

Transcription is an important biological activity in a cell as it is the first step for the transmission of genetic information from DNA into RNA to be translated into proteins [47]. With the suppression of the essential transcriptional proteins for eukaryotic chromosomal DNA replication such as DNA-directed RNA polymerase II 16kDa polypeptide, transcription elongation factor B polypeptide 1, and proliferating cell nuclear antigen, transcription initiation and elongation process becomes inefficient [48,49]. Besides that, zinc finger protein 174 is a DNA binding protein that acts as a cofactor for transcription factor. Its downregulation will lead to increased transcription of proteins such as E-cadherin which in turn represses cell invasion [50]. Also, cell cycle disorder plays a critical role in cancer progression. So, modulation of cell cycle by phytochemicals from natural product sources is gaining worldwide attention to control carcinogenesis [51]. However, our findings showed that cell cycle pathway was not modulated with the flow cytometric data showing insignificant shifts in each cell cycle phases for the cells treated with *Phyllanthus* extracts. Hence, cell cycle arrest was ruled out as one of the mechanism of actions of the extracts. Since *Phyllanthus* do not inflict cell cycle arrest on A549, the only approach to inhibit A549′s continuous growth is by causing toxicity. As showed in the previous study, *Phyllanthus* does induce apoptosis in A549 with more than three folds increase of caspases-3 and -7, the presence of DNA-fragmentation and TUNEL-positive cells [17]. In order to determine whether *Phyllanthus* induces extrinsic or intrinsic apoptotic pathway, Bcl-2 expression was examined using immunoblot analysis since it is one of the main regulators of the mitochondrial outer membrane permeabilization which initiates intrinsic apoptotic cell death [52,53]. The data obtained agrees with our previous hypothesis that *Phyllanthus* probably activates the intrinsic pathway of apoptosis by inhibiting antiapoptotic Bcl-2 protein to release cytochrome c for caspases activation. In addition to this, proteomic analysis also observed downregulation of Bcl-2-like protein 11 which is similar to Bcl-2 protein that has a role as an antiapoptotic protein [52]. Apart from that, suppressing MAP kinases and its downstream factors such as AP-1 have been shown to decrease MMPs expression and subsequently inhibit various pathological processes such as tumor invasion, adhesion, metastasis, and angiogenesis [22,24]. This is in accordance with results from the current study that showed decreased ERK1/2 pathway and MMPs activity by *Phyllanthus* which led to inhibition of A549 metastasis. Moreover, *Phyllanthus* extracts also suppressed cytoskeletal proteins such as actin, vimentin, tubulin alpha chain, actin-related protein, and cofilin-1/2. Besides being the components of the cytoskeleton, both actin and tubulin-binding proteins are also mediators of motility [8]. Structure, conformational dynamics, and mechanical properties of actin filaments are mainly controlled by cofilin [54]. Meanwhile, vimentin constitutes the intermediate filaments of the cytoskeleton which stabilizes cytoskeletal interactions as well as affecting cell motility and movement. Elevated expression of vimentin in several invasive cell lines suggests the possibility of it being a representative marker for epithelial to mesenchymal transition [38]. Therefore, the significant inhibitory effects of *Phyllanthus* on the A549 cell’s cytoskeleton most probably involve the alteration of the microfilament organization and function, therefore suppressing motility and metastasis [55].

Hypoxia is largely perceived as another major obstacle to cancer therapy as increasing evidence in the cancer therapy-related literatures suggests the involvement of proangiogenic factors in the progression of lung tumorigenesis [7]. Angiogenesis is essential for tumor growth and survival due to the imbalance of nutrient and oxygen supplies to solid tumors larger than 1 mm^3^, resulting in tumor hypoxia [56]. One of the major regulatory component which responds to hypoxia to ensure cell survival and to promote angiogenesis is HIF-1α [2,7]. Therefore, it could possibly be the target for the development of novel anticancer agents. Lin et al. [7] demonstrated suppression of lung tumor angiogenesis and metastasis by andrographolide isolated from *Andrographis paniculata* which downregulates HIF-1α. In addition, inhibition of HIF-1α pathway by *HIF-1α–siRNA* displayed a direct correlation with A549 cellular proliferation and angiogenesis, a prerequisite for metastasis [26]. In agreement to these studies, our results obtained also showed the reduction of HIF-1α expression in A549 cells treated with *Phyllanthus* extracts. This in turn led to the suppression of various target genes controlled by HIF-1α via hypoxia-responsive-element (HRE) [26], including angiogenesis, metabolism, cell growth, and death [2].

VEGF is another crucial angiogenic growth factor which induces endothelial cell proliferation from the pre-existing capillary bed for wound healing, tumor growth, and metastasis. Its expression is therefore increased prior to an invasive and metastatic phenotype [56]. Also iNOS, one of the three distinct isoforms of NOS which is widely expressed and often upregulated in multiple tumor tissues [57] is expressed in tumor cells associated with vascularization and hence, is probably another important regulator of angiogenesis [56,57]. Nitric oxide (NO) produced by NOS have been shown to affect vascular permeability, induce extracellular matrix degradation, trigger VEGF production, as well as stimulate endothelial cell proliferation and migration [30]. Hence, inhibition of VEGF and iNOS by *Phyllanthus* can greatly reduce A549 angiogenesis, resulting in tumor cells malnutrition and hypoxia thereby preventing tumor growth, survival, and metastasis. Adenosine 5′-triphosphate, a major source of energy for cells and its involvement in a variety of cellular activities which are ATP-dependent is often increased in tumor cells [58]. Downregulation of these enzymes activities by *Phyllanthus* causes cellular energy deficit that can result in cancer cell death. One example is enolase which catalyzes conversion of 2-phosphoglyceric acid (PGA) to phosphoenolpyruvate (PEP) in the anabolic pathway during gluconeogenesis to enhance aerobic glycolysis in cancer cells [59]. Other enzymes suppressed include glyceraldehyde 3 phosphate dehydrogenase, fructose-biphosphate aldolase A, phosphoglycerate mutase 1, and triosephosphate isomerase that are also involved in energy metabolism [55,60].

Besides exploiting cellular signaling pathways for their growth and metastasis, tumor cells possess efficient drug detoxification system to remove compounds that may be fatal to them. This includes upregulation of glutathione transferase omega-1 that catalyzes binding of glutathione to various anticancer compounds such as cisplatin, thereby decreasing production of platinum-DNA adducts and rendering them useless while glutathione synthetase catalyzes production of glutathione substrate for the detoxifying activity [61,62]. Similarly, metallothionein also plays a role in chemotherapy binding and detoxification since its elevated expression was noticed in several cisplatin-resistant lung cancer cell lines [61]. Meanwhile, Annexin A4 is normally associated with chemoresistance in part by enhancing drug efflux [63]. Inhibition of these detoxification enzymes expressions in A549 after treatment with *Phyllanthus* as demonstrated from the proteomic analysis therefore advocates the reduced A549 drug-resistance capability resulting in their susceptibility to death-inducing compounds.

## Conclusions

All the findings obtained in this study point to the involvement of ERK1/2 and hypoxia pathways which were suppressed by *Phyllanthus* to inhibit A549 proliferation, angiogenesis, invasion, and metastasis. Inhibition of ERK1/2 pathway led to downregulation of invasion and mobility proteins (MMP2; MMP7; MMP9; cytoskeletal proteins), transcriptional proteins (proliferating cell nuclear antigen; zinc finger protein), and antiapoptotic protein (Bcl2) while inhibition of hypoxia pathway causes repression of angiogenic proteins (VEGF; iNOS) and various glycolytic enzymes. Suppression of drug detoxification enzymes such as gluthathione transferase and metallothionein also increases sensitivity of A549 to *Phyllanthus* treatment. Among the four *Phyllanthus* species tested in this study, *P. urinaria* was found to be the most effective to inhibit A549 growth and metastasis, closely followed by *P. watsonii*. Thus, *Phyllanthus* could be a valuable candidate in the treatment of metastatic cancers. However, the main concern before application of *Phyllanthus* as an antimetastatic or antiproliferative agent is its *in vivo* effect. Thus, further testing of the extracts activity *in vivo* is necessary to exploit it as a chemotherapeutic agent. Preliminary work has identified low toxicity of *Phyllanthus* in an animal model as dosage greater than 50 g/kg is needed to cause 100% acute death in the mice tested (unpublished data) and subsequent efficacy testing in a tumor-bearing mice model needs to be carried out.

## Competing interests

The authors declare that they have no competing interests.

## Authors’ contributions

SDS conceived of the study, participated in its design and coordination as well as helped to draft and edited the manuscript. SHL participated in the design of the study, carried out the experimental works, performed the statistical analysis, and drafted the manuscript. IBJ carried out extracts preparation and edited the manuscript. RM involved in early conception of study design, clinical coordination, manuscript editing of the clinical components and proof reading. All authors read and approved the final manuscript.

## Pre-publication history

The pre-publication history for this paper can be accessed here:

http://www.biomedcentral.com/1472-6882/13/271/prepub

## References

[B1] Luo X Liu Y Wang R Hu H Zeng R Chen H A high-quality secretome of A549 cells aided the discovery of C4b-binding protein as a novel serum biomarker for non-small cell lung cancer J Proteomics 2011 13 4 528 538 10.1016/j.jprot.2011.01.011 21262398

[B2] Ye MX Zhao YL Li Y Miao Q Li ZK Ren XL Song LQ Yin H Zhang J Curcumin reverses cis-platin resistance and promotes human lung adenocarcinoma A549/DDP cell apoptosis through HIF-1alpha and caspase-3 mechanisms Phytomedicine 2012 13 8–9 779 787 22483553 10.1016/j.phymed.2012.03.005

[B3] Li B Chang J Chu Y Kang H Yang J Jiang J Ma H Membrane proteomic analysis comparing squamous cell lung cancer tissue and tumour-adjacent normal tissue Cancer Lett 2012 13 1 118 124 10.1016/j.canlet.2011.12.037 22252117

[B4] Ko JC Tsai MS Weng SH Kuo YH Chiu YF Lin YW Curcumin enhances the mitomycin C-induced cytotoxicity via downregulation of MKK1/2-ERK1/2-mediated Rad51 expression in non-small cell lung cancer cells Toxicol Appl Pharmacol 2011 13 3 327 338 10.1016/j.taap.2011.07.012 21810436

[B5] Yao H Zhang Z Xiao Z Chen Y Li C Zhang P Li M Liu Y Guan Y Yu Y Identification of metastasis associated proteins in human lung squamous carcinoma using two-dimensional difference gel electrophoresis and laser capture microdissection Lung Cancer 2009 13 1 41 48 10.1016/j.lungcan.2008.10.024 19058872

[B6] Guo F Hiroshima K Wu D Satoh M Abulazi M Yoshino I Tomonaga T Nomura F Nakatani Y Prohibitin in squamous cell carcinoma of the lung: its expression and possible clinical significance Hum Pathol 2012 13 8 1282 1288 10.1016/j.humpath.2011.10.006 22304787

[B7] Lin HH Tsai CW Chou FP Wang CJ Hsuan SW Wang CK Chen JH Andrographolide down-regulates hypoxia-inducible factor-1alpha in human non-small cell lung cancer A549 cells Toxicol Appl Pharmacol 2011 13 3 336 345 10.1016/j.taap.2010.11.014 21134392

[B8] Murphy L Henry M Meleady P Clynes M Keenan J Proteomic investigation of taxol and taxotere resistance and invasiveness in a squamous lung carcinoma cell line Biochim Biophys Acta 2008 13 9 1184 1191 10.1016/j.bbapap.2008.04.014 18503785

[B9] Karpova MA Moshkovskii SA Toropygin IY Archakov AI Cancer-specific MALDI-TOF profiles of blood serum and plasma: biological meaning and perspectives J Proteomics 2010 13 3 537 551 10.1016/j.jprot.2009.09.011 19782778

[B10] Lim R Lappas M Ahmed N Permezel M Quinn MA Rice GE 2D-PAGE of ovarian cancer: analysis of soluble and insoluble fractions using medium-range immobilized pH gradients Biochem Biophys Res Commun 2011 13 3 408 413 10.1016/j.bbrc.2011.02.056 21329656

[B11] Fekkar A Pionneau C Brossas JY Marinach-Patrice C Snounou G Brock M Ibrahim-Granet O Mazier D DIGE enables the detection of a putative serum biomarker of fungal origin in a mouse model of invasive aspergillosis J Proteomics 2012 13 9 2536 2549 10.1016/j.jprot.2012.01.040 22370163

[B12] Fujita Y Nakanishi T Miyamoto Y Hiramatsu M Mabuchi H Miyamoto A Shimizu A Takubo T Tanigawa N Proteomics-based identification of autoantibody against heat shock protein 70 as a diagnostic marker in esophageal squamous cell carcinoma Cancer Lett 2008 13 2 280 290 10.1016/j.canlet.2008.01.013 18334280

[B13] Rodriguez-Pineiro AM Blanco-Prieto S Sanchez-Otero N Rodriguez-Berrocal FJ de la Cadena MP On the identification of biomarkers for non-small cell lung cancer in serum and pleural effusion J Proteomics 2010 13 8 1511 1522 10.1016/j.jprot.2010.03.005 20230924

[B14] Afjehi-Sadat L Shin JH Felizardo M Lee K Slavc I Lubec G Detection of hypothetical proteins in 10 individual human tumor cell lines Biochim Biophys Acta 2005 13 1 67 80 10.1016/j.bbapap.2004.09.024 15680240

[B15] Wong PF Cheong WF Shu MH Teh CH Chan KL AbuBakar S Eurycomanone suppresses expression of lung cancer cell tumor markers, prohibitin, annexin 1 and endoplasmic reticulum protein 28 Phytomedicine 2012 13 2 138 144 10.1016/j.phymed.2011.07.001 21903368

[B16] Paranjpe P Indian medicinal plants : forgotten healers : a guide to ayurvedic herbal medicine with identity, habitat, botany, photochemistry, ayurvedic properties, formulations & clinical usage 2001 Delhi Varanasi: Chaukhamba Sanskrit Pratishthan; Also available at Chaukhamba Surbharati Prakashan 48 50

[B17] Lee SH Jaganath IB Wang SM Sekaran SD Antimetastatic effects of Phyllanthus on human lung (A549) and breast (MCF-7) cancer cell lines PLoS One 2011 13 6 e20994 10.1371/journal.pone.0020994 21698198 PMC3116853

[B18] Joshi H Parle M Brahmi rasayana improves learning and memory in mice Evid Based Complement Alternat Med 2006 13 1 79 85 10.1093/ecam/nek014 16550227 PMC1375237

[B19] Lee SH Tang YQ Rathkrishnan A Wang SM Ong KC Manikam R Payne BJ Jaganath IB Sekaran SD Effects of cocktail of four local Malaysian medicinal plants (Phyllanthus spp.) against dengue virus 2 BMC Complement Altern Med 2013 13 1 192 10.1186/1472-6882-13-192 23889893 PMC3726501

[B20] Tang YQ Jaganath IB Sekaran SD Phyllanthus spp. induces selective growth inhibition of PC-3 and MeWo human cancer cells through modulation of cell cycle and induction of apoptosis PLoS One 2010 13 9 e12644 10.1371/journal.pone.0012644 20838625 PMC2935893

[B21] Shih YW Shieh JM Wu PF Lee YC Chen YZ Chiang TA alpha-Tomatine inactivates PI3K/Akt and ERK signaling pathways in human lung adenocarcinoma A549 cells: effect on metastasis Food Chem Toxicol 2009 13 8 1985 1995 10.1016/j.fct.2009.05.011 19457446

[B22] Chen PN Hsieh YS Chiou HL Chu SC Silibinin inhibits cell invasion through inactivation of both PI3K-Akt and MAPK signaling pathways Chem-Biol Interact 2005 13 2–3 141 150 16169542 10.1016/j.cbi.2005.08.005

[B23] Chen YY Chou PY Chien YC Wu CH Wu TS Sheu MJ Ethanol extracts of fruiting bodies of Antrodia cinnamomea exhibit anti-migration action in human adenocarcinoma CL1-0 cells through the MAPK and PI3K/AKT signaling pathways Phytomedicine 2012 13 8–9 768 778 22464013 10.1016/j.phymed.2012.02.016

[B24] Hsiao YC Kuo WH Chen PN Chang HR Lin TH Yang WE Hsieh YS Chu SC Flavanone and 2′-OH flavanone inhibit metastasis of lung cancer cells via down-regulation of proteinases activities and MAPK pathway Chem-Biol Interact 2007 13 3 193 206 10.1016/j.cbi.2007.02.012 17376416

[B25] Liu D Nakano J Ishikawa S Yokomise H Ueno M Kadota K Urushihara M Huang CL Overexpression of matrix metalloproteinase-7 (MMP-7) correlates with tumor proliferation, and a poor prognosis in non-small cell lung cancer Lung Cancer 2007 13 3 384 391 10.1016/j.lungcan.2007.07.005 17728005

[B26] Hanze J Eul BG Savai R Krick S Goyal P Grimminger F Seeger W Rose F RNA interference for HIF-1alpha inhibits its downstream signalling and affects cellular proliferation Biochem Biophys Res Commun 2003 13 3 571 577 10.1016/j.bbrc.2003.10.153 14680803

[B27] Teijido O Dejean L Upregulation of Bcl2 inhibits apoptosis-driven BAX insertion but favors BAX relocalization in mitochondria FEBS Lett 2010 13 15 3305 3310 10.1016/j.febslet.2010.07.002 20621101

[B28] Weber A Kristiansen I Johannsen M Oelrich B Scholmann K Gunia S May M Meyer HA Behnke S Moch H The FUSE binding proteins FBP1 and FBP3 are potential c-myc regulators in renal, but not in prostate and bladder cancer BMC Cancer 2008 13 369 10.1186/1471-2407-8-369 19087307 PMC2631590

[B29] Xu K Liang X Gao F Zhong J Liu J Antimetastatic effect of ganoderic acid T in vitro through inhibition of cancer cell invasion Process Biochem 2010 13 8 1261 1267 10.1016/j.procbio.2010.04.013

[B30] Cullis ER Kalber TL Ashton SE Cartwright JE Griffiths JR Ryan AJ Robinson SP Tumour overexpression of inducible nitric oxide synthase (iNOS) increases angiogenesis and may modulate the anti-tumour effects of the vascular disrupting agent ZD6126 Microvasc Res 2006 13 2 76 84 10.1016/j.mvr.2006.01.004 16530791

[B31] Nalivaeva NN Turner AJ Post-translational modifications of proteins: acetylcholinesterase as a model system Proteomics 2001 13 6 735 747 10.1002/1615-9861(200106)1:6<735::AID-PROT735>3.0.CO;2-8 11677779

[B32] Walsh G Jefferis R Post-translational modifications in the context of therapeutic proteins Nat Biotechnol 2006 13 10 1241 1252 10.1038/nbt1252 17033665

[B33] Ruvinsky I Meyuhas O Ribosomal protein S6 phosphorylation: from protein synthesis to cell size Trends Biochem Sci 2006 13 6 342 348 10.1016/j.tibs.2006.04.003 16679021

[B34] Martineau Y Derry MC Wang X Yanagiya A Berlanga JJ Shyu AB Imataka H Gehring K Sonenberg N Poly(A)-binding protein-interacting protein 1 binds to eukaryotic translation initiation factor 3 to stimulate translation Mol Cell Biol 2008 13 21 6658 6667 10.1128/MCB.00738-08 18725400 PMC2573229

[B35] Khalil AA Kabapy NF Deraz SF Smith C Heat shock proteins in oncology: Diagnostic biomarkers or therapeutic targets? Biochim Biophys Acta 2011 13 2 89 104 21605630 10.1016/j.bbcan.2011.05.001

[B36] Justice SS Hunstad DA Harper JR Duguay AR Pinkner JS Bann J Frieden C Silhavy TJ Hultgren SJ Periplasmic peptidyl prolyl cis-trans isomerases are not essential for viability, but SurA is required for pilus biogenesis in Escherichia coli J Bacteriol 2005 13 22 7680 7686 10.1128/JB.187.22.7680-7686.2005 16267292 PMC1280321

[B37] Yoo BC Fountoulakis M Dierssen M Lubec G Expression patterns of chaperone proteins in cerebral cortex of the fetus with Down syndrome: dysregulation of T-complex protein 1 J Neural Transm Suppl 2001 13 321 334 11771755 10.1007/978-3-7091-6262-0_27

[B38] Walsh N O’Donovan N Kennedy S Henry M Meleady P Clynes M Dowling P Identification of pancreatic cancer invasion-related proteins by proteomic analysis Proteome Sci 2009 13 3 10.1186/1477-5956-7-3 19216797 PMC2646716

[B39] Li S Shang Y Regulation of SRC family coactivators by post-translational modifications Cell Signal 2007 13 6 1101 1112 10.1016/j.cellsig.2007.02.002 17368849

[B40] Crow T Xue-Bian JJ Proteomic analysis of post-translational modifications in conditioned Hermissenda Neuroscience 2010 13 4 1182 1190 10.1016/j.neuroscience.2009.11.066 19961907 PMC2815081

[B41] Tuteja N Signaling through G protein coupled receptors Plant Signal Behav 2009 13 10 942 10.4161/psb.4.10.9530 19826234 PMC2801357

[B42] Cross TG Scheel-Toellner D Henriquez NV Deacon E Salmon M Lord JM Serine/threonine protein kinases and apoptosis Exp Cell Res 2000 13 1 34 41 10.1006/excr.2000.4836 10739649

[B43] Nishida E Gotoh Y The MAP kinase cascade is essential for diverse signal transduction pathways Trends Biochem Sci 1993 13 4 128 10.1016/0968-0004(93)90019-J 8388132

[B44] Hindley A Kolch W Extracellular signal regulated kinase (ERK)/mitogen activated protein kinase (MAPK)-independent functions of Raf kinases J Cell Sci 2002 13 Pt 8 1575 1581 11950876 10.1242/jcs.115.8.1575

[B45] Roberts PJ Der CJ Targeting the Raf-MEK-ERK mitogen-activated protein kinase cascade for the treatment of cancer Oncogene 2007 13 22 3291 3310 10.1038/sj.onc.1210422 17496923

[B46] Bachmann M Moroy T The serine/threonine kinase Pim-1 Int J Biochem Cell Biol 2005 13 4 726 730 10.1016/j.biocel.2004.11.005 15694833

[B47] Solomon EP Berg LR Martin DW Biology 2008 8 Australia, United States: Thomson-Brooks/Cole 318 319

[B48] Naryzhny SN Proliferating cell nuclear antigen: a proteomics view Cell Mol Life Sci 2008 13 23 3789 3808 10.1007/s00018-008-8305-x 18726183 PMC11131649

[B49] Scicchitano DA Transcription past DNA adducts derived from polycyclic aromatic hydrocarbons Mutat Res 2005 13 1–2 146 154 15922365 10.1016/j.mrfmmm.2005.03.015

[B50] Comijn J Berx G Vermassen P Verschueren K van Grunsven L Bruyneel E Mareel M Huylebroeck D van Roy F The two-handed E box binding zinc finger protein SIP1 downregulates E-cadherin and induces invasion Mol Cell 2001 13 6 1267 1278 10.1016/S1097-2765(01)00260-X 11430829

[B51] Abdolmohammadi MH Sh F Shafiee A Gh A Ghaffari SM Azizi E Anticancer effects and cell cycle analysis on human breast cancer T47D cells treated with extracts of Astrodaucus persicus (Boiss.) Drude in comparison to doxorubicin DARU J Pharm Sci 2008 13 2 112 118

[B52] Brunelle JK Letai A Control of mitochondrial apoptosis by the Bcl-2 family J Cell Sci 2009 13 Pt 4 437 441 19193868 10.1242/jcs.031682 PMC2714431

[B53] Chipuk JE Green DR How do BCL-2 proteins induce mitochondrial outer membrane permeabilization? Trends Cell Biol 2008 13 4 157 164 10.1016/j.tcb.2008.01.007 18314333 PMC3242477

[B54] Pfaendtner J De La Cruz EM Voth GA Actin filament remodeling by actin depolymerization factor/cofilin Proc Natl Acad Sci USA 2010 13 16 7299 7304 10.1073/pnas.0911675107 20368459 PMC2867716

[B55] Fang HY Chen SB Guo DJ Pan SY Yu ZL Proteomic identification of differentially expressed proteins in curcumin-treated MCF-7 cells Phytomedicine 2011 13 8–9 697 703 21239154 10.1016/j.phymed.2010.11.012

[B56] Dachs GU Tozer GM Hypoxia modulated gene expression: angiogenesis, metastasis and therapeutic exploitation Eur J Cancer 2000 13 13 Spec No 1649 1660 10959051 10.1016/s0959-8049(00)00159-3

[B57] Fitzpatrick B Mehibel M Cowen RL Stratford IJ iNOS as a therapeutic target for treatment of human tumors Nitric Oxide 2008 13 2 217 224 10.1016/j.niox.2008.05.001 18515106

[B58] Shigenaga MK Hagen TM Ames BN Oxidative damage and mitochondrial decay in aging Proc Natl Acad Sci USA 1994 13 23 10771 10778 10.1073/pnas.91.23.10771 7971961 PMC45108

[B59] Liu KJ Shih NY The role of enolase in tissue invasion and metastasis of pathogens and tumor cells J Cancer Mol 2007 13 2 45 48

[B60] Ruiz-Romero C Carreira V Rego I Remeseiro S Lopez-Armada MJ Blanco FJ Proteomic analysis of human osteoarthritic chondrocytes reveals protein changes in stress and glycolysis Proteomics 2008 13 3 495 507 10.1002/pmic.200700249 18186017

[B61] Stewart DJ Tumor and host factors that may limit efficacy of chemotherapy in non-small cell and small cell lung cancer Crit Rev Oncol Hematol 2010 13 3 173 234 10.1016/j.critrevonc.2009.11.006 20047843 PMC2888634

[B62] Polekhina G Board PG Gali RR Rossjohn J Parker MW Molecular basis of glutathione synthetase deficiency and a rare gene permutation event EMBO J 1999 13 12 3204 3213 10.1093/emboj/18.12.3204 10369661 PMC1171401

[B63] Kim A Enomoto T Serada S Ueda Y Takahashi T Ripley B Miyatake T Fujita M Lee CM Morimoto K Enhanced expression of Annexin A4 in clear cell carcinoma of the ovary and its association with chemoresistance to carboplatin Int J Cancer 2009 13 10 2316 2322 10.1002/ijc.24587 19598262

